# Native mass spectrometric studies of IscSU reveal a concerted, sulfur-initiated mechanism of iron–sulfur cluster assembly†

**DOI:** 10.1039/d2sc04169c

**Published:** 2022-11-15

**Authors:** Sophie P. Bennett, Jason C. Crack, Rita Puglisi, Annalisa Pastore, Nick E. Le Brun

**Affiliations:** Centre for Molecular and Structural Biochemistry, School of Chemistry, University of East Anglia Norwich Research Park Norwich NR4 7TJ UK j.crack@uea.ac.uk n.le-brun@uea.ac.uk; The Wohl Institute, King's College London, Denmark Hill Campus London SE5 8AF UK

## Abstract

Iron–sulfur (Fe–S) clusters are cofactors essential for life. Though the proteins that function in the assembly of Fe–S clusters are well known, details of the molecular mechanism are less well established. The Isc (iron–sulfur cluster) biogenesis apparatus is widespread in bacteria and is the closest homologue to the human system. Mutations in certain components of the human system lead to disease, and so further studies of this system could be important for developing strategies for medical treatments. We have studied two core components of the Isc biogenesis system: IscS, a cysteine desulfurase; and IscU, a scaffold protein on which clusters are built before subsequent transfer onto recipient apo-proteins. Fe^2+^-binding, sulfur transfer, and formation of a [2Fe–2S] was followed by a range of techniques, including time-resolved mass spectrometry, and intermediate and product species were unambiguously identified through isotopic substitution experiments using ^57^Fe and ^34^S. Under cluster synthesis conditions, sulfur adducts and the [2Fe–2S] cluster product readily accumulated on IscU, but iron adducts (other than the cluster itself) were not observed at physiologically relevant Fe^2+^ concentrations. Our data indicate that either Fe^2+^ or sulfur transfer can occur first, but that the transfer of sulfane sulfur (S^0^) to IscU must occur first if Zn^2+^ is bound to IscU, suggesting that it is the key step that initiates cluster assembly. Following this, [2Fe–2S] cluster formation is a largely concerted reaction once Fe^2+^ is introduced.

## Abstract 2

Time-resolved native mass spectrometry was used to investigate iron–sulfur cluster assembly on IscU. Data revealed a concerted assembly process in which sulfur (S^0^) transfer must occur first if IscU is in its Zn^2+^-bound form.

## Graphical abstract

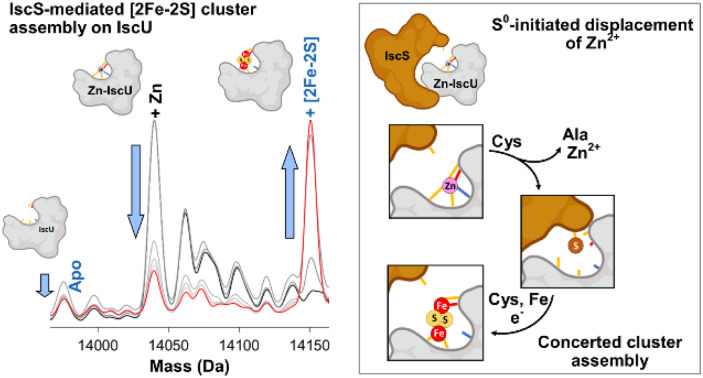

## Introduction

A diverse range of cellular processes, including respiration, central metabolism and DNA repair, rely on proteins containing an iron–sulfur (Fe–S) cluster.^1^ This versatile prosthetic group can perform multiple functions including in electron transport, transcriptional regulation, redox catalysis, and sulfur donation.^1,2^ As well as being essential for virtually all bacteria, including pathogens, an increasing number of human diseases are now recognised as being associated with impaired maturation of Fe–S proteins.^3,4^ Amongst these is the neurodegenerative disorder Friedrich's ataxia, in which a component of the Fe–S cluster biogenesis machinery (frataxin) is deficient or carries mutations that alter its function^5^ or stability,^6^ resulting in impaired maturation of respiratory complexes I–III.^7^ An understanding of this machinery in both prokaryotes and eukaryotes is therefore essential.

In bacteria, three well-known systems are responsible for Fe–S cluster assembly and post-translational insertion into apo-proteins: the *isc* and *suf* operons encode housekeeping assembly machineries that generate Fe–S clusters under normal and stress conditions, respectively.^8,9^ The *nif* operon encodes the system responsible for Fe–S cluster maturation specific to nitrogenase. The Isc assembly machinery, as exemplified by that encoded by the *iscRSUA-hscBA-fdx* operon in *E. coli*, is the most widespread system, and has direct orthologues in the eukaryotic mitochondrion.^10^

IscS and IscU are two essential components of the Isc Fe–S cluster biogenesis apparatus in *E. coli*. IscS is a cysteine desulfurase with a covalently attached pyridoxal phosphate (PLP) cofactor, which converts l-cysteine to alanine, generating sulfane sulfur (S^0^).^11,12^ Homologues of IscS are found in both Suf and Nif systems. The IscS desulfurase in *E. coli* has a more general role, producing S^0^ for other processes in addition to Fe–S cluster biogenesis. For example, TusA accepts S^0^ from IscS and supplies it to a range of biosynthetic pathways, such as molybdenum cofactor biosynthesis and thiomodifications of tRNA.^13–15^

**Fig. 1:**
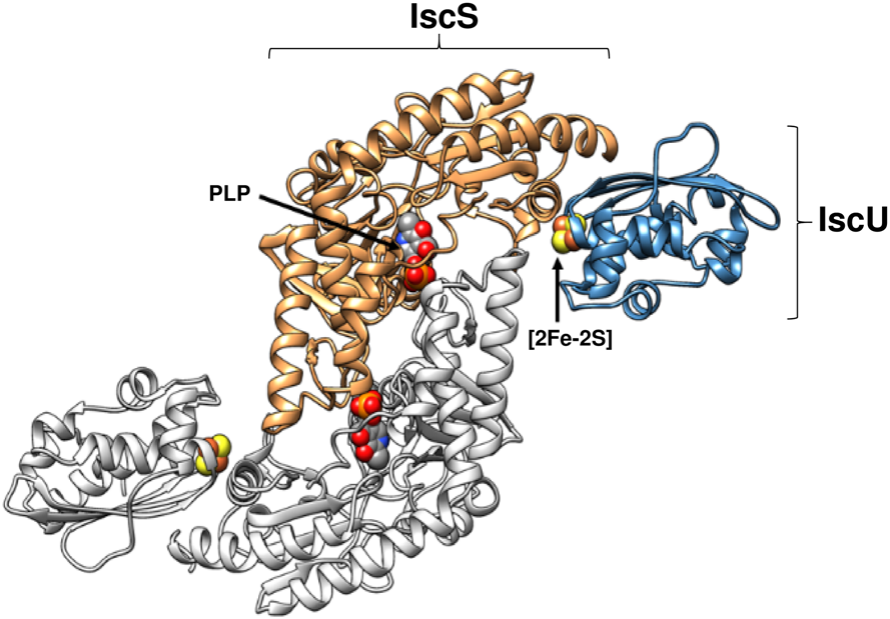
Structure of (IscS)_2_(IscU)_2_ complex. Ribbon diagram representing the structure of *Archaeoglobus fulgidus* (IscS)_2_(IscU)_2_, with PLP and [2Fe–2S] cluster cofactors indicated in space filling mode (PDB 4EB5).

The cysteine-derived S^0^ is stored on a conserved cysteine residue in IscS (Cys328 in the *E. coli* protein), generating a cysteine persulfide, and, in partnership with the scaffold protein IscU, a [2Fe–2S] cluster is assembled on IscU.^16–19^ A high resolution structure of IscS and IscU in complex revealed a heterotetramer consisting of a dimeric IscS core with two IscU proteins associated at the polar ends,^17^ see Fig. 1. The *in vitro* reconstitution of a [2Fe–2S] cluster on IscU from a reaction mixture containing IscS, cysteine, iron and reducing equivalents has been demonstrated.^16,20,21^ The *in vivo* source of iron for the cluster is unclear.^22–25^ IscX and CyaY (the bacterial orthologue of the eukaryotic protein frataxin) bind iron and associate with the same surface on IscS. The affinities of these proteins for IscS depend on the iron concentration, leading to the conclusion that accessible iron within the cell regulates Fe–S cluster biogenesis.^22,26^ A ferredoxin, Fdx, is also encoded by the isc operon and likely contributes the reducing equivalents needed to release sulfur from IscS to the cluster as sulfide (S^2−^).^27,28^

The IscS-mediated assembly of a cluster on IscU is the starting point of a process by which Fe–S cluster proteins are matured. The subsequent steps involve transfer of the assembled [2Fe–2S] cluster from the scaffold to recipient apo-proteins. Additional proteins, such as the transfer proteins IscA and ErpA, and the chaperones HscA and HscB,^9,29–32^ are involved in this. Alternatively, the [2Fe–2S] cluster bound form of IscU can undergo a reductive coupling transformation, to generate a [4Fe–4S] cluster bound to a IscU dimer.^33^

IscU is isolated as a variable mixture of apo- and Zn^2+^-bound forms.^34–36^ Since Zn^2+^ and iron–sulfur clusters share similar coordinating ligands (*e.g.* Cys, His, Asp) in proteins, it has been proposed that zinc directly disrupts preassembled Fe–S clusters of the dehydratase class of enzymes (*e.g.* aconitase, fumarase).^37^ Like iron, the amount of ‘free’ intracellular zinc in bacteria is tightly regulated, and for Zn^2+^ rarely exceeds picomolar levels due to its toxic effects. Although the physiological role of Zn^2+^–IscU in *E. coli* remains unclear, binding appears to be tight (estimates of the *K*_d_ for the Zn^2+^–IscU complex range from micromolar^38^ to sub-picomolar^34^), and so it is possible that Zn^2+^–IscU may be relevant *in vivo* under certain conditions. Indeed, Li *et al.* recently demonstrated that zinc specifically inhibits IscU/IscA-mediated Fe–S assembly and transfer, rather than disrupting already assembled clusters.^39^

The precise nature of the events leading to the formation of a cluster on IscU, including Zn^2+^ removal, Fe^2+^ and sulfur delivery/insertion, and intermediates formed along the way to the final product have yet to be clearly established. Native (non-denaturing) mass spectrometry, in which non-covalent interactions are preserved, can provide high resolution mass data for protein–protein and protein–cofactor interactions^40–44^ and has been established recently as a powerful tool for investigating the Fe–S cluster machinery and elucidating chemistry involving protein-bound Fe–S clusters.^45–51^ Recently, native mass spectrometry was applied to Fe–S biogenesis through studies of the IscSU complex, leading to the identification of intermediate species and the proposal of an overall ‘iron-first’ mechanism.^52^

Here, through the application of native mass spectrometry together with other biophysical techniques, we provide further insight into the cluster assembly process involving IscS and IscU, by addressing questions relating to: the conformational flexibility of IscU, and its interaction with IscS; Fe^2+^- and S^0^-binding to IscU, and the importance of the presence of Zn^2+^ on IscU; the possibility of detecting intermediate Fe/S species during cluster assembly on IscU. Our data support the dynamic interconversion of IscU conformational states in the absence of IscS, and the effect of bound Zn^2+^ ions on this equilibrium. We also determine affinities of IscU for Fe^2+^ and for IscS, and demonstrate that Zn^2+^ binding prevents Fe^2+^-binding to IscU, thus inhibiting FeS assembly. Furthermore, the removal of Zn^2+^ requires IscS-mediated S^0^ transfer, and the ensuing mechanism of IscS-mediated [2Fe–2S] cluster assembly on IscU is largely concerted, with no significant accumulation of intermediates.

## Materials and methods

### Protein production

All proteins used in this study are from *E. coli*. Their corresponding DNA sequences were subcloned into a pET-24d vector, encoding fusion proteins with His-tagged glutathione-*S*-transferase (GST), and used to transform *E. coli* BL21 DE3. Cultures for the overproduction of IscU were grown in Luria Broth enriched medium containing 8.3 mg per L ZnSO_4_ (ref. 20) to help stabilize the folded form. IscS was expressed in the presence of 100 μM PLP (Sigma). The proteins were purified by affinity chromatography using a His-Trap column (GE Healthcare) and cleaved from the His-GST tag by tobacco virus protease (TEV) protease. The cleaved protein solution was reloaded onto a His-Trap column to separate the His-tagged GST from IscU.^20^

Protein purity was confirmed by SDS-PAGE and liquid chromatography mass spectrometry (LC-MS). Protein concentrations were determined by absorbance at 280 nm using *ε*_280 nm_ of 41 370 or 11 460 M^−1^ cm^−1^ for IscS and IscU, respectively. IscU was treated with either a 25-fold excess of diethylene triamine pentaacetate (DTPA) to chelate Zn^2+^,^53^ or a 2-fold excess of ZnSO_4_ to produce a fully zinc-loaded form of IscU,^34^ before exchanging the protein into 50 mM Tris, 150 mM NaCl, pH 8.0 using a desalting column (PD10, Cytiva). Metal ion contents of IscU samples were determined by inductively coupled plasma-mass spectrometry (ICP-MS) using an iCAP-TQ (Thermofisher Scientific) instrument. Aerobically purified IscU contained Zn^2+^ (∼0.5 per protein), as previously observed,^34,36^ while DTPA-treated IscU contained ∼0.01 Zn^2+^ per protein.

For LC-MS, a 20 μM aliquot of protein was diluted with an aqueous mixture of 2% (v/v) acetonitrile, 0.1% (v/v) formic acid, and loaded onto a Proswift RP-1S column (4.6 × 50 mm, Thermo Scientific) attached to an Ultimate 3000 uHPLC system (Dionex, Leeds, UK) with eluant infused into the electrospray ionisation (ESI) source of a Bruker micrOTOF-QIII mass spectrometer (Bruker Daltonics) operating in the positive ion mode. Processing and analysis of MS experimental data were carried out using Compass DataAnalysis v4.1 (Bruker Daltonik). Neutral mass spectra were generated using the ESI Compass v1.3 Maximum Entropy deconvolution algorithm. LC-MS of purified isolated IscU gave a mass of 13 974 Da, which corresponds to the predicted mass of the apo-protein with a −2 Da mass shift, indicating that Zn^2+^ was lost under the denaturing conditions of the LC-MS experiment, and an intra-molecular disulfide bond had formed (see Table 1).

**Table 1:** Predicted and observed masses of Isc proteins

Protein species	Predicted mass (Da)	Average observed mass^a^ (Da)	ΔMass^b^ (Da)	^57^Fe and ^34^S isotope shift (Da)
**LC-MS**				
IscU	13 976	13 974	−2	
IscS	45 288	45 288	0	

**Native MS**				
IscU (aerobic)	13 976	13 974	−2	
IscU (anaerobic)	13 976	13 976	0	
(IscU)_2_	27 952	27 946	−6	
Zn^2+^–IscU	14 039	14 040	+1	
Fe^2+^–IscU	14 030	14 030	0	
Zn^2+^–, Fe^2+^–IscU	14 092	14 093	+1	
(Fe^2+^)_2_–IscU	14 084	14 084	0	
[2Fe–2S]–IscU	14 150	14 151	+1	S: +4
				Fe: +2
S–IscU	14 008	14 007	−1	S: +2
(S)_2_–IscU	14 040	14 040	0	S: +2
(S)_3_–IscU	14 072	14 072	0	S: +4
(S)_4_–IscU	14 104	14 104	0	S: +6
Fe^3+^–, S^2−^–IscU	14 061	14 061	0	
Fe^2+^–, S^2−^–, S^0^–IscU	14 094	14 094	0	
Zn^2+^–, S–IscU	14 070	14 071	+1	
Zn^2+^–, (S)_2_–IscU	14 102	14 103	+1	
SO_4_^2−^–IscU	14 074	14 075	+1	—
Zn^2+^–, SO_4_^2−^–IscU	14 137	14 138	+1	—
(IscS)_2_ (with PLP bound)	91 038	91 036	−2	—

a The average observed mass is derived from at least three independent experiments, with standard deviation of ±1 Da.

b The difference between the average observed and predicted masses.

### Cluster reconstitution on IscU

All enzymatic IscU Fe–S cluster reconstitution experiments were performed in an anaerobic chamber (Belle technology) under nitrogen atmosphere (O_2_ < 2 ppm). Catalytic amounts of IscS were initially used because at higher concentrations, sulfur adducts of +32 Da accumulated on IscU to an extent that made cluster assembly much more difficult to follow. IscU concentrations were kept below 15 μM because higher concentrations were found to block the nebulizer needle when conducting native mass spectrometry. Hence, optimised reaction mixtures contained 0.2 μM IscS, 10 μM IscU, 20 μM ferrous ammonium sulfate, 50 μM l-cysteine and 2 mM DTT, unless otherwise stated. Isotopic substitution experiments were conducted using ^34^S-cysteine and ^57^Fe, as previously reported.^46,54^ The use of ammonium acetate as volatile buffer was found to inhibit cluster reconstitution, possibly due to competition for Fe^2+^-binding, or for the cysteine binding site on IscS, and so ammonium formate was used instead. Cluster assembly was also followed by measuring *A*_456 nm_ as a function of time using a Jasco V550 spectrophotometer,^20,21^ or by circular dichroism measurements made using a Jasco J-810 CD spectropolarimeter.^55^

The effect on cluster assembly of pre-loading of IscU with S^0^ or Fe^2+^, or Zn^2+^/Fe^2+^, was investigated. Apo-IscU (∼200 μM) containing S^0^ was prepared as previously described.^56^ Briefly, IscU was treated with 10 μM IscS and 3 mM l-cysteine under aerobic conditions and incubated at 37 °C for 1 h, prior to desalting (PD10, Cytiva). S^0^ loading was confirmed by routine LC-MS. Fe^2+^-loading was achieved by treating apo-IscU with a 30-fold excess of ferrous ammonium sulfate under anaerobic conditions. The solution was incubated at an ambient temperature for 15 min, prior to desalting (PD10, Cytiva). For sequential Zn^2+^/Fe^2+^ treatment, IscU was first treated with a 30-fold excess of zinc sulfate in the presence of 3 mM DTT under anaerobic conditions. The resulting solution was incubated at an ambient temperature for 15 min, prior to desalting (PD10, Cytiva). The sample was then treated with a 30-fold excess of ferrous ammonium sulfate, as described above, then desalted.

IscU treated with S^0^, Fe^2+^ or sequential Zn^2+^/Fe^2+^ was then used as the sole source of sulfur or iron for *in vitro* Fe–S assembly. Where IscU (∼50 μM) served as the sulfur source, Fe–S assembly was initiated following the addition of 0.17 mM ferrous ammonium sulfate in the presence of absence of 2 mM DTT, GSH, or l-cysteine. Where IscU (∼50 μM) served as the iron source, Fe–S assembly was initiated following the addition of 5 μM IscS and 2 mM l-cysteine. The buffer was 100 mM Tris, pH 7.5. Fe–S cluster assembly was followed by UV-visible absorbance. IscU-bound Fe^2+^ was determined *via* Ferrene™ (Merck)^57^ and bound Zn^2+^ was determined *via* 4-(2-pyridylazo)resorcinol (Merck).^58^

For non-denaturing (native) mass spectrometry measurements, Isc proteins were buffer exchanged at room temperature in an anaerobic chamber into ammonium formate or ammonium acetate, volatile buffers that assist with desolvation of proteins as they transition into the gas phase. Samples were then loaded into a 500 μL gas-tight syringe and infused directly in a Bruker micrOTOF-QIII mass spectrometer (Bruker Daltonics) operating in the positive ion mode. The ESI-TOF was calibrated online using ESI-L Low Concentration Tuning Mix (Agilent Technologies) and, where necessary, subsequently re-calibrated offline in the 4000–8000 *m*/*z* range using a 4.6 mM caesium perfluoroheptanoate mix. Data were acquired over the *m*/*z* range of 600–3000 for IscU with acquisition controlled by Bruker qTOFControl software, with parameters as follows: dry gas flow 4 L min^−1^, nebulise gas pressure 0.8 Bar, dry gas 180 °C, capillary voltage 3000 V, offset 500 V, quadrupole voltage 5 V, collision RF 650 Vpp. The *m*/*z* acquired data were averaged over the given time period and the neutral mass spectrum calculated using the ESI Compass v1.3 Maximum Entropy (MaxEnt) deconvolution algorithm, taking into account all charge states detected in the 600–3000 *m*/*z* region. Average resolution for this range was 20 000 FWHM. For IscS and IscS complexes, a *m*/*z* range of 4000–8000 was used to acquire data, with parameters as follows: dry gas flow 4 L min^−1^, nebulise gas pressure 0.8 Bar, dry gas 180 °C, capillary voltage 3000 V, offset 500 V, quadrupole voltage 5 V, collision RF 2650 Vpp, collision cell voltage 10 eV, isCID energy 120 eV. Deconvolution was performed as described above but over the mass range 4000–8000 *m*/*z*. Average resolution for this range was 10 500 FWHM. Isotopic exchange experiments with ^57^Fe and ^34^S were performed as previously described.^45,54^^34^S was supplied as ^34^S-cysteine, synthesised using a thermostable cysteine synthase as previously described.^54^

### Determination of Fe^2+^-binding affinity *via* competition assay

The dissociation constant (*K*_d_) for Fe^2+^-binding to apo-IscU was determined using an Fe^2+^-binding competition assay employing the divalent metal ligand mag-fura-2, as previously described.^48,59^ Mag-fura-2 forms a 1 : 1 complex with Fe^2+^, resulting in a shift of absorbance maximum from 366 nm (metal free) to 325 nm (Fe^2+^ bound).^59^ Briefly, apo-IscU (at ∼5 μM or 12 μM) was mixed with 5 μM mag-fura-2 in 1 mL of buffer (20 mM HEPES, 150 mM NaCl, pH 7.5, or 200 mM ammonium formate, pH 8.0) under anaerobic conditions. Incremental amounts of Fe^2+^ (as ferrous ammonium sulfate) were added until binding saturation was achieved. Binding data were simultaneously fitted with Dynafit using eqn (1) and (2):1
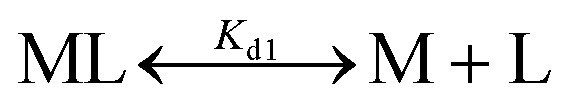
2
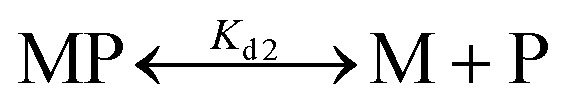
where M is Fe^2+^, P is IscU, and L is mag-fura-2. Concentrations of L and ML were determined directly from the response of mag-fura-2 at *A*_366 nm_ and *A*_325 nm_, respectively. As a control, mag-fura-2 in 200 mM ammonium formate, pH 8.0 was titrated with Fe^2+^, to determine any effect of formate ions, which could compete for Fe^2+^.

### The effect of ESI on the Fe^2+^-binding affinity of IscU

Solutions of apo-IscU (15.5 μM), in 200 mM ammonium formate, 0.94 mM DTT, pH 8, were mixed with increasing concentrations of iron(ii) formate (0–100 IscU/Fe^2+^ molar ratios). Samples were loaded into a 500 μL gas-tight syringe (Hamilton) and infused directly into the ionisation source of the mass spectrometer. Data were acquired (750–4000 *m*/*z* region) for 5 min, averaged, and the neutral mass spectrum calculated (9000–38 000 Da). For the determination of apparent dissociation constants, ion counts for Fe^2+^–IscU were compared with the combined ion counts for apo-IscU, and IscU +32 Da (S or 2O), +64 Da (Zn), and +54 Da (Fe). At least two datasets were acquired, expressed as fractional saturation, then averaged. The response of Fe^2+^–IscU in the average data set was fitted using eqn (2), from which an apparent *K*_d_ for Fe^2+^ binding under conditions of ESI-MS was estimated. A stock solution of iron(ii) formate (67 mM) was prepared under anaerobic conditions by reacting ferrous ammonium sulfate with excess sodium bicarbonate, resulting in Fe(ii)CO_3_ precipitate. This was isolated, reacted with formic acid, and diluted in 200 mM ammonium formate pH 8.0. The solution of formate was further diluted to 1.7 mM just prior to use.

### IscS-free reconstitution of Fe–S–IscU

The lack of cluster intermediate species during IscS-catalysed reconstitutions, might have been due to a component of the reaction mixture affecting ionisation; thus, we sought to simplify the reconstitution by pre-generating a source of S^0^. 23.4 mM elemental sulfur (S^0^) was solubilised overnight in 2 mL of 200 mM ammonium acetate pH 7.0 containing 36.6 mM DTT and 200 mM NH_4_OH (final ratio of S^0^ to DTT, 1 : 1.6).^60–62^ The resulting solution was used as a low molecular weight IscS mimic (DTT-S), see Scheme S1.† To initiate reconstitution, IscU (10.5 μM) in 200 mM ammonium formate pH 8.0 was pre-treated with a 54-fold excess of DTT-S (0.56 mM, final conc.) for ∼50 s and then, immediately before infusion, the sample was treated with an ∼18-fold excess (0.18 mM, final conc.) of (NH_4_)_2_Fe(ii)(SO_4_)_2_ in DTT (1.8 mM, final conc.) and mixed. Data were acquired from ∼20 s post iron addition for up to 5 min, averaged (every 0.3 min), and the neutral mass spectrum calculated (9000–38 000 Da). Zn^2+^–IscU served as an internal standard and the ion counts combined with those for apo-IscU, and IscU +32 Da (S), +54 Da (Fe), +86 Da (Fe–S), +108 Da (Fe_2_), +118 Da (Fe–S_2_), +153 Da (DTT), +174 Da (Fe_2_S_2_). At least two datasets were acquired, expressed as fractional saturation, and then averaged.

### IscS–IscU interaction measurements

To evaluate the *K*_d_ of the IscSU complex by native mass spectrometry, an aliquot of purified protein (as isolated IscU or IscS) was first exchanged into 250 mM ammonium acetate pH 8.0 using desalting columns (PD MiniTrap G25, Cytiva). The protein concentration was determined by absorbance and working solutions (200 μL) were prepared by combining aliquots of IscS and IscU to achieve the desired concentration or IscS/IscU ratio, then infused directly into the source of a Waters Synapt XS (Waters Inc.) mass spectrometer operating in the positive ion mode. Data were acquired and processed with Mass Lynx v4.2 (Waters Inc.) over the *m*/*z* range of 3000 to 8000 *m*/*z* with parameters as follows: dry gas flow 4 L min^−1^, nebuliser gas pressure 6 Bar, dry gas 150 °C, source temperature 80 °C, capillary voltage 3100 V, offset 30 V, cone voltage (isCID) 150 V, trap collisional energy 10 eV, and transfer collision energy 2 eV. Spectra were averaged, and the neutral mass spectrum calculated (90–125 kDa) using the MaxEnt1 deconvolution algorithm of MassLynx v4.2 (Waters Inc.). At least two datasets were acquired, expressed as fractional saturation, and then averaged prior to fitting with Dynafit (Biokin), as previously described.^22^ The mass spectrometer was calibrated with sodium iodide. Average resolution for the measured range was 20 000 FWHM.

## Results

### IscU exists as an equilibrium between folded and disordered states in the absence of IscS

**Fig. 2:**
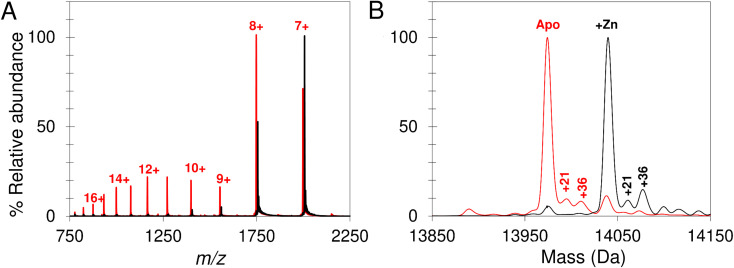
Apo- and Zn^2+^–IscU have different charge distributions. The acquired *m*/*z* spectrum (A) and deconvoluted neutral mass spectrum (B) for apo-IscU (<1% Zn^2+^, in red) and Zn–IscU (100% Zn^2+^, black) by non-denaturing mass spectrometry, in 200 mM ammonium formate, pH 8.0. Charge states for IscU are noted in red (A). Adducts masses, noted in Daltons, correspond to Na (+21) and Cl or Na/O (+36) (B).

Native mass spectrometry was used to study IscU. Mass spectra for apo-IscU and Zn^2+^–IscU under aerobic conditions are shown in Fig. 2. Zn^2+^–IscU exhibited two dominant charge states of 7+ and 8+, which were also present in the spectrum of apo-IscU, along with a second envelope of charge states at lower *m*/*z* values (charge states 9+ to 17+, Fig. 2A). This indicated the presence of two conformational states of this form of the protein. Deconvolution across all charge states gave a spectrum with apo-IscU as the major species (at a mass of 13 974 Da, corresponding to oxidised IscU containing one disulfide bond). The two apo-IscU charge state envelopes were also deconvoluted separately (Fig. S1†), revealing that the more highly charged ions arise from apo-IscU, while the 7+ and 8+ charge states arise from apo-IscU and the small component of Zn^2+^-bound IscU present in the sample.

The similarity between the higher charge state envelop and that observed using LC-MS (where the protein was unfolded), indicates that this form of the protein is less well folded. This is also consistent with the tendency of folded proteins to exhibit lower charge states compared to unfolded forms because fewer amino acid residues are available for protonation. In addition to monomeric oxidised IscU, a dimeric form of apo-IscU was also observed by native MS, containing an inter-molecular disulfide (Fig. S2A†). Addition of excess DTT and measurement after 1 min revealed the almost complete loss of the dimeric form, and an increase in reduced monomeric IscU in the D state (Fig. S2B†). Re-measurement after 5 min indicated the re-equilibration of the sample with an increase in S state relative to D state monomeric IscU (Fig. S2C and D†).

**Fig. 3:**
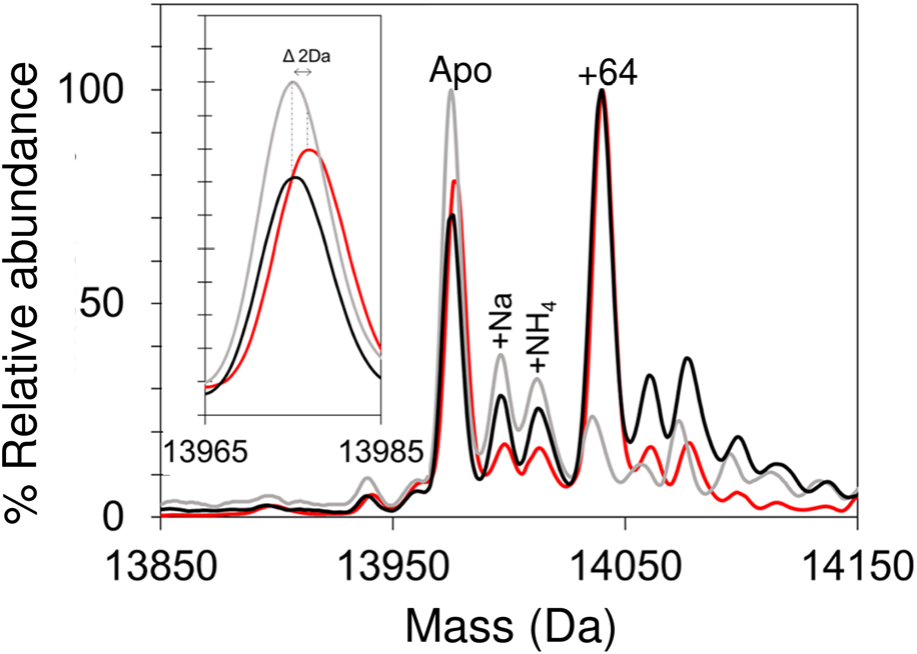
Anaerobic/aerobic cycling of IscU modulates Zn^2+^-binding. Deconvoluted neutral mass spectrum of apo-IscU prepared under anaerobic conditions (red), exposed to atmospheric O_2_ for 4 hours (grey) and made anaerobic again by incubation in an anaerobic chamber for 2 h (black). Inset is a plot of the apo-IscU mass region, illustrating that a −2 Da shift occurs open exposure to O_2_, which is reversed upon return to anaerobic conditions.

Apo-IscU was then prepared under anaerobic conditions (in preparation for cluster reconstitution) and native MS recorded. Surprisingly, a peak at +64 Da was found to dominate the deconvoluted mass spectrum, which could arise from either Zn^2+^ or two sulfurs bound to IscU. Reducing agents such as DTT or TCEP typically remove sulfur adducts but, in this case, there was no effect on the +64 Da adduct. Furthermore, LC-MS did not reveal any significant covalent adducts of IscU (sulfur adducts, multiples of +32 Da, are covalently attached as persulfides and survive LC-MS conditions).^50,51,63^ Thus, we conclude that the IscU +64 Da peak is not due to sulfur, and must be due to Zn^2+^, even though analysis had demonstrated that the protein contained ≤0.01 Zn^2+^ per protein (Fig. 3).

The unexpected prevalence of the Zn^2+^-bound form of IscU in the spectrum of apo-IscU under anaerobic conditions, compared to what was observed under aerobic conditions (Fig. 2), suggested that the redox state of the protein could be important. Thus, an anaerobically treated IscU sample was exposed to air for 4 h and the native mass spectrum re-measured (Fig. 3). Following introduction of air, the peak due to apo-IscU was observed to be the only major species. Exposure of Zn^2+^-loaded IscU to air had no effect on the mass spectrum, suggesting that the effect is confined to samples principally containing apo-IscU.

The O_2_-exposed IscU sample was then returned to the anaerobic glove box for 2 h (Fig. 3); the resulting deconvoluted native mass spectrum was similar to that recorded initially for the anaerobic sample, with the Zn^2+^-form once again the more prevalent. The mass of the apo-IscU peak following O_2_ exposure (13 974 Da) indicated oxidation of the protein *via* the introduction of one disulfide bond, resulting in a −2 Da mass shift. Removal of O_2_ essentially restored the peak to the position of fully reduced IscU (see inset Fig. 3). These data illustrate that these different forms of IscU can be cycled.

Further evidence that the adduct is due to Zn^2+^ was derived from the *m*/*z* spectrum of the aerobic, oxidised sample (corresponding to a mix of S and D states), for which charge states between 500 and 2500 *m*/*z* were observed, while under anaerobic conditions the most intense peaks were the 7+ and 8+ charge states (corresponding to the S state).

To rationalise the above observations, it is important to acknowledge that mass spectrometry under native conditions is not a quantitative technique in terms of comparing signal intensities between two species. This is because of the variability in ionisation efficiency, where one species may ionise much more readily than another. The IscU data could arise from the much more efficient ionisation of the Zn^2+^-bound form relative to the apo-form, or more specifically, reduced apo-IscU. For closely related species, very different ionisation efficiencies would be unusual. In this case, it is known that Zn^2+^-binding to IscU stabilizes the structured state, and so the changes in IscU due to Zn^2+^-binding are more significant than only the binding of a metal ion. The formation of an intra-molecular disulfide bond upon exposure to O_2_ could stabilise IscU.

Another possibility is that reduced IscU, which binds Zn^2+^ with high affinity,^34^ is able to scavenge Zn^2+^ from the anaerobic glove box environment, or from buffer components/DTT (*e.g.* ref. 64). In such a situation, the apparent loss of Zn^2+^–IscU upon exposure to O_2_ could result from displacement of Zn^2+^ upon oxidation of coordinating Cys thiols to a disulfide.^65^ However, similar anaerobic/aerobic experiments with IscU containing 0.5 or 1 Zn^2+^ per protein did not result in significant changes in the spectrum, indicating that displacement of Zn^2+^ upon exposure of IscU to O_2_ does not alone account for the observed mass changes above. Thus, the behaviour of IscU-derived peaks in native MS spectra upon redox cycling most likely arises from the suppression of reduced IscU ionisation by Zn^2+^–IscU in combination with some degree of scavenging of Zn^2+^.

Overall, we conclude that in mixtures of reduced, apo-IscU and Zn^2+^–IscU, native MS signals due to the latter are more intense than predicted from its solution abundance. Both structured (S) and disordered (D) conformational states of IscU are present under the conditions of the native mass spectrometry experiment. Zn^2+^–IscU is structured, while apo-IscU exists as an equilibrium mixture of S and D forms, consistent with previous studies that showed that Zn^2+^ stabilises the folded conformation of IscU.^34,36,66^

### Fe^2+^-binding to IscU

**Fig. 4:**
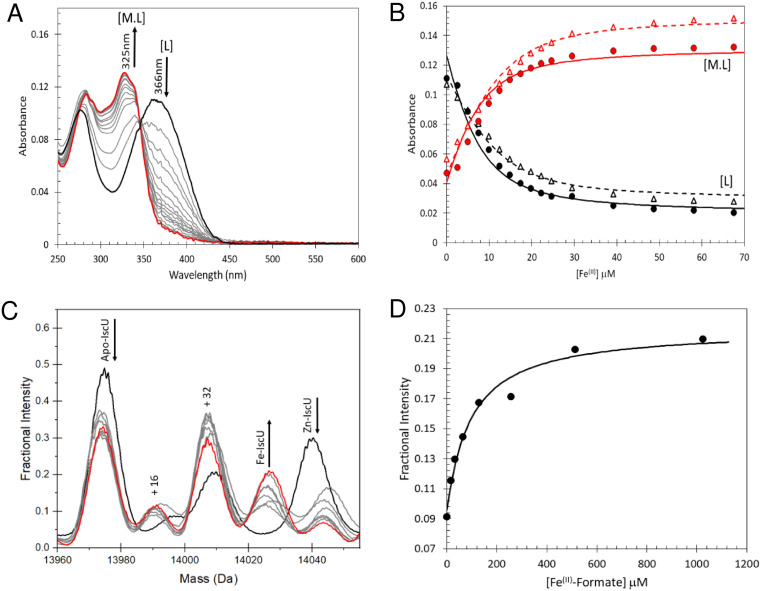
Determination of the binding affinity of Fe^2+^–IscU. (A) Spectra showing Fe^2+^ binding to mag-fura-2 (5 μM) in competition with apo-IscU (5 μM) in 20 mM HEPES, 150 mM NaCl pH 7.5, leading to a transition from apo- (*λ*_max_ 366 nm) to Fe^2+^–mag-fura-2 (*λ*_max_ 325 nm). Arrows indicate direction of absorbance changes for apo- and Fe^2+^–mag-fura-2, [L] and [ML], respectively. An equivalent titration of mag-fura-2 alone with Fe^2+^ was performed as a control experiment from which the affinity of Fe^2+^ for mag-fura-2 was confirmed as *K*_d_ = 2.0 (±0.2) μM. (B) Absorbance changes at measured at 325 nm (red points) and 366 nm (black points) following the addition of increasing concentrations of Fe^2+^ to mag-fura-2 in competition with 5.0 μM (closed circles, solid lines) and 12.4 μM (open triangles, dashed lines). The data were simultaneously fitted in Dynafit using a simple binding equation, see Results, giving an apparent *K*_d_ of 3.5 (±0.2) μM for IscU. (C) Deconvoluted mass spectrum of apo-IscU (9 μM) titrated with Fe^2+^–formate and ionised from 200 mM ammonium formate pH 8. We note that a proportion of apo-IscU remains at the end of the titration in an oxidised form that appears unable to bind Fe^2+^. The species at 14 044 Da is unknown. (D) Plot of fractional intensity due to Fe^2+^–IscU as a function of Fe^2+^–formate added. The dissociation constant for Fe^2+^–IscU under these conditions was *K*_d_ ∼ 73 μM.

To determine the stability of Fe^2+^–IscU, we used the well characterised Fe^2+^–chelator mag-fura-2 in a competition assay (Fig. 4A), in which binding was monitored by optical titration, as previously described.^48,59^ (Fig. 4B). The resulting dissociation constants for Fe^2+^–mag-fura-2 and Fe^2+^–IscU were *K*_d_ = 2.0 ± 0.2 μM and *K*_d_ = 3.5 ± 0.2 μM (Fig. 4B), respectively, in close agreement with previously published binding constants for mag-fura-2 and IscU from prokaryotic or eukaryotic sources.^59,67–69^

Next, we sought to determine the affinity of apo-IscU for Fe^2+^ under native MS conditions, to evaluate whether the presence of high concentrations of formate interferes with Fe^2+^-binding (Fig. S3†). Thus, similar experiments as those above were performed, except that 200 mM ammonium formate pH 8.0 was used. No effect of changing the buffer was observed when Fe^2+^ was titrated in to ammonium formate solutions containing mag-fura-2 alone (Fig. S3A and B†), or containing apo-IscU (Fig. S3C–F†), confirming that formate does not compete for Fe^2+^ under these conditions.

We then attempted to measure Fe^2+^-binding directly using native MS. Here, apo-IscU in ammonium formate was titrated with increasing amounts of Fe^2+^, and the formation of Fe^2+^–IscU followed by native MS (Fig. 4C). The apparent affinity constant for Fe^2+^–IscU under these conditions was determined as *K*_d_ = 73 ± 13 μM (Fig. 4D), ∼20-fold lower affinity than that measured by in solution competition assay (see above). Thus, while there are many examples in which behaviour of proteins in the gas phase closely mimics that in solution, in this case there is a clear effect on the affinity of IscU for Fe^2+^ in the gas phase. The consequence of this is that it is more difficult than expected to observe Fe^2+^-bound forms of IscU by native MS.

**Fig. 5:**
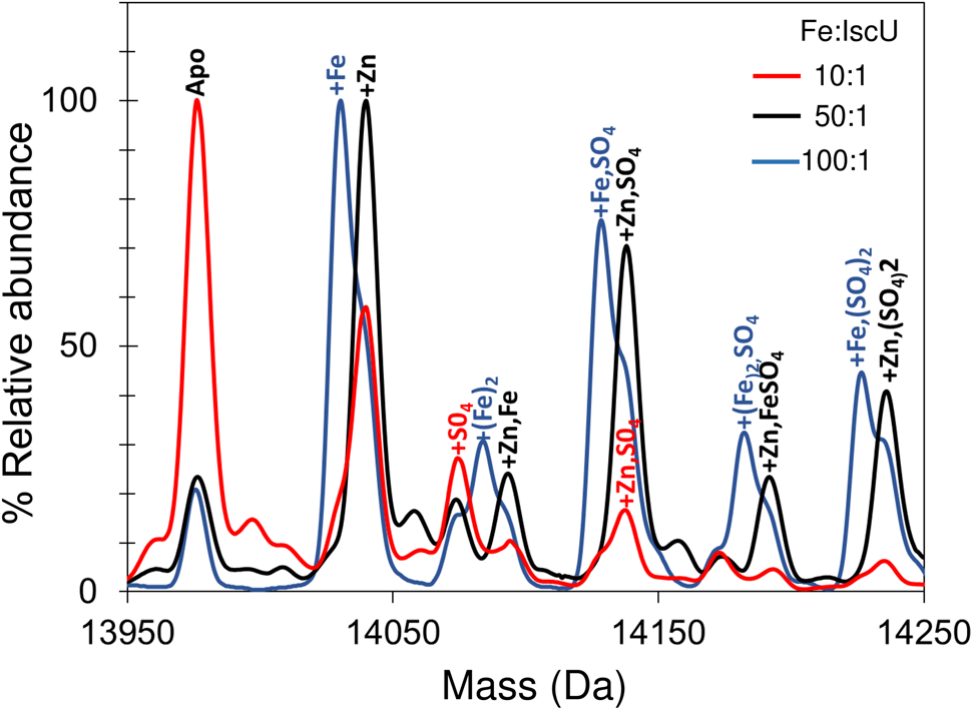
Iron binding to IscU. Incubation of IscU in 200 mM ammonium formate, with 2 mM DTT and a 5, 50 and 100-fold excess of ferrous ammonium sulfate. Fe^2+^-bound forms of IscU, along with sulfate adducts, are detected.

Addition of a 10-fold excess of Fe^2+^ over apo-IscU resulted in a small peak that could be due to Zn^2+^/Fe^2+^-bound IscU, along with sulfate adducts (Fig. 5). At a 50-fold excess of iron, the peak due to a possible Zn^2+^/Fe^2+^ adduct was much more clearly resolved, while at a 100-fold excess of iron, IscU species with one and two irons bound (at 14 030 and 14 083 Da, respectively) were observed along with a loss of the Zn^2+^/Fe^2+^ peak. The *m*/*z* spectrum of IscU with a 100-fold excess of Fe^2+^ contained the lower charge states (7+, 8+) only, indicating stabilization of IscU in the S state by Fe^2+^-binding (Fig. S4†).

### Interactions between IscS and IscU

**Fig. 6:**
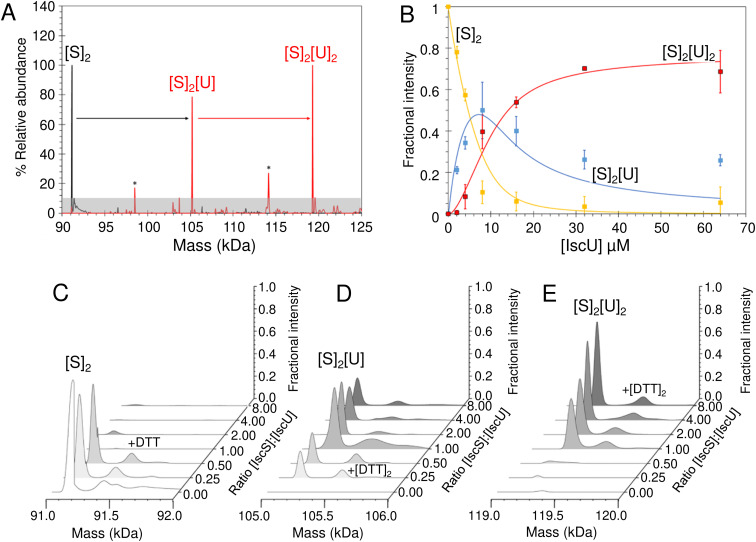
ESI-MS investigation of complex formation between IscS and IscU. (A) Deconvoluted mass spectra of IscS over the mass range 90–125 kDa, showing the presence of the IscS dimer (black spectrum). Addition of IscU at a 2 : 1 excess (as indicated) gave rise to a series of IscU–IscS complexes in which the IscS dimer is bound by 1–2 IscU molecules. Asterisks indicate unknown species that may be due to complexes of IscS with degraded forms of IscU. (B) Plots of fractional intensity of the two IscU–IscS complexes, as indicated, as a function of IscU concentration. Solid lines show fits of the data to a sequential binding model for 1–2 IscU per IscS dimer. The binding of one or two IscU molecules to dimeric IscS occurred with a similar affinity, *K*_d_ of 3.26 ± 0.46 μM. (C)–(E) Representative deconvoluted mass spectra at increasing ratios of IscU to IscS showing the formation/decay of the IscU–IscS complexes, as indicated. An adduct species (+154 or +308 Da) is present in each of the spectra, including that of the IscS dimer, indicating that it originates from IscS. The precise nature of the adduct is unknown, but it is likely to arise from β-mercaptoethanol or DTT hetero-disulfides with IscS, as previously observed.^22^ IscS (8 μM) and IscU were in 250 mM ammonium acetate pH 8.

We then looked into the interactions between IscU and IscS. The *K*_d_ of the IscU–IscS complex in solution has been determined to be 1–2 μM by isothermal calorimetry,^19,70^ consistent with the dynamic role of these two proteins play in Fe–S cluster assembly (IscU–IscS) and Fe–S delivery (IscU). Under native MS conditions, the *m*/*z* spectrum for as isolated IscS (Fig. S5†) contained four principal charge states (17+ to 20+), which upon deconvolution gave a mass of 91 036 Da, in close agreement with that expected for a dimer of IscS with PLP bound to each protomer (Fig. S5B,†Table 1). Addition to IscS of a 2-fold excess of IscU resulted in significant changes in the *m*/*z* spectrum. The deconvoluted spectrum (spanning 90–125 kDa) revealed the presence of a new species, corresponding to dimeric IscS in complex with up to two IscU molecules in their structured form, Fig. 6A.

Titration of IscS with increasing concentrations of as isolated IscU resulted in the gradual formation of dimeric IscS complex containing 1 or 2 IscU molecules ((IscS)_2_(IscU) or (IscS)_2_(IscU)_2_, Fig. 6B–E). (IscS)_2_(IscU) formed readily at low levels of IscU (IscU/IscS ≈ 0.5) and maximised at IscU/IscS ≈ 1.0, with the concomitant loss of un-complexed dimeric IscS. The (IscS)_2_(IscU)_2_ complex was evident at IscU/IscS ≈ 1.0 and became the dominant species at an IscU/IscS ≈ 8.0 Fig. 6C–E. The data were analysed according to a sequential binding model. The resulting data fit revealed that binding of one or two IscU molecules to dimeric IscS occurred with a similar affinity, *K*_d_ = ∼3 μM, consistent with solution studies (Fig. 6B).^70^ As the binding behaviour is well described by a single average dissociation constant, there is no evidence for cooperativity, suggesting that the IscS dimer contains two equivalent but independent binding sites for IscU.

To investigate if IscS had a preference for apo-IscU or Zn^2+^–IscU, they were separately added to IscS and native mass spectrometry measurements performed at fixed IscS–IscU ratios. Deconvoluted spectra revealed that both monomeric apo- and Zn^2+^-forms of IscU interact with the IscS dimer to form either (IscS)_2_IscU and/or (IscS)_2_(IscU)_2_ complexes (Fig. S6†). We also investigated whether the presence of IscS influences the binding of Fe^2+^ to IscU. A 100-fold excess of iron (as described above) was added to apo-IscU in the presence of IscS, both at catalytic concentration and at a 2 : 1 ratio of IscU : IscS (but in the absence of substrate cysteine). Up to two irons per IscU monomer were observed (Fig. S7†), as in the absence of IscS, indicating that the presence of IscS does not significantly affect the affinity of IscU for iron. Thus, the stability of the IscS–IscU complex(es) and binding of metal ions on IscU do not significantly affect one another. Similarly, binding of IscU to one IscS of the dimer does not affect binding of IscU at the other.

### Reconstitution of [2Fe–2S] cluster on IscU monitored by native mass spectrometry

**Fig. 7:**
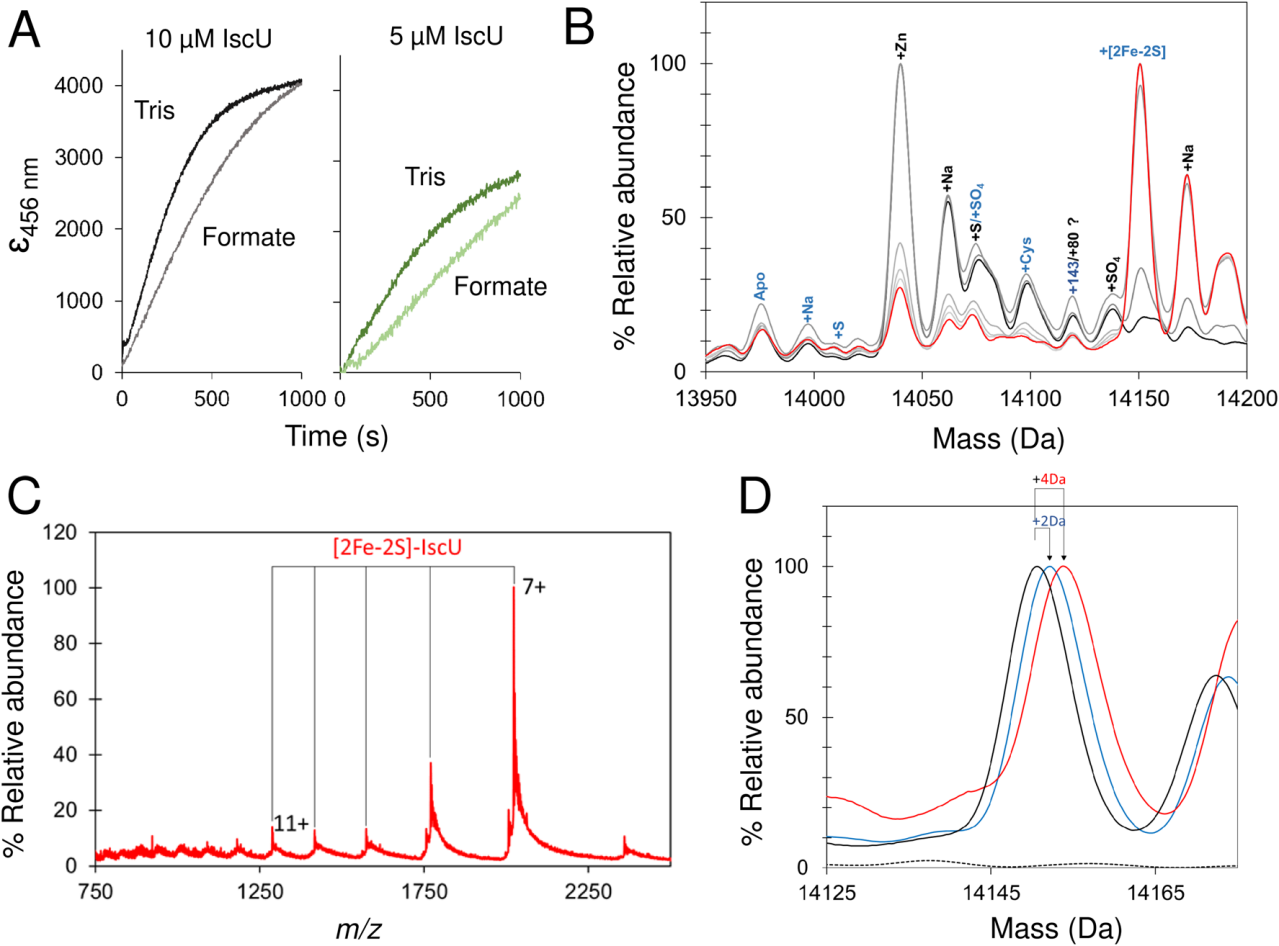
Synthesis of a [2Fe–2S] cluster on IscU. (A) Reconstitution of apo-IscU (10 or 5 μM, as indicated) in 50 mM Tris, 150 mM NaCl, pH 8.0, (darker green) or 200 mM ammonium formate pH 8.0 (lighter green), followed at 456 nm and plotted as *ε*_456 nm_ to account for protein concentration difference. (B) Time-resolved deconvoluted mass spectra of the reconstitution reaction in the IscU region measured over 25 min. Starting and final spectra are in black and red, respectively, with intervening spectra in grey. (C) *m*/*z* spectrum measured after 25 min of reconstitution of IscU (containing 0.01 Zn^2+^ per protein) in 200 mM ammonium formate buffer, pH 8.0 with 10 μM IscU, 20 μM Fe, 50 μM cysteine, 2 mM DTT and 0.2 μM IscS. Charge states due to [2Fe–2S] IscU indicated. (D) The [2Fe–2S] peak for IscU reconstituted using natural abundance cysteine and iron (black spectrum), ^57^Fe (blue) and ^34^S Cys (red). Mass shifts due to the incorporation of ^57^Fe and ^34^S are indicated.

**Fig. 8:**
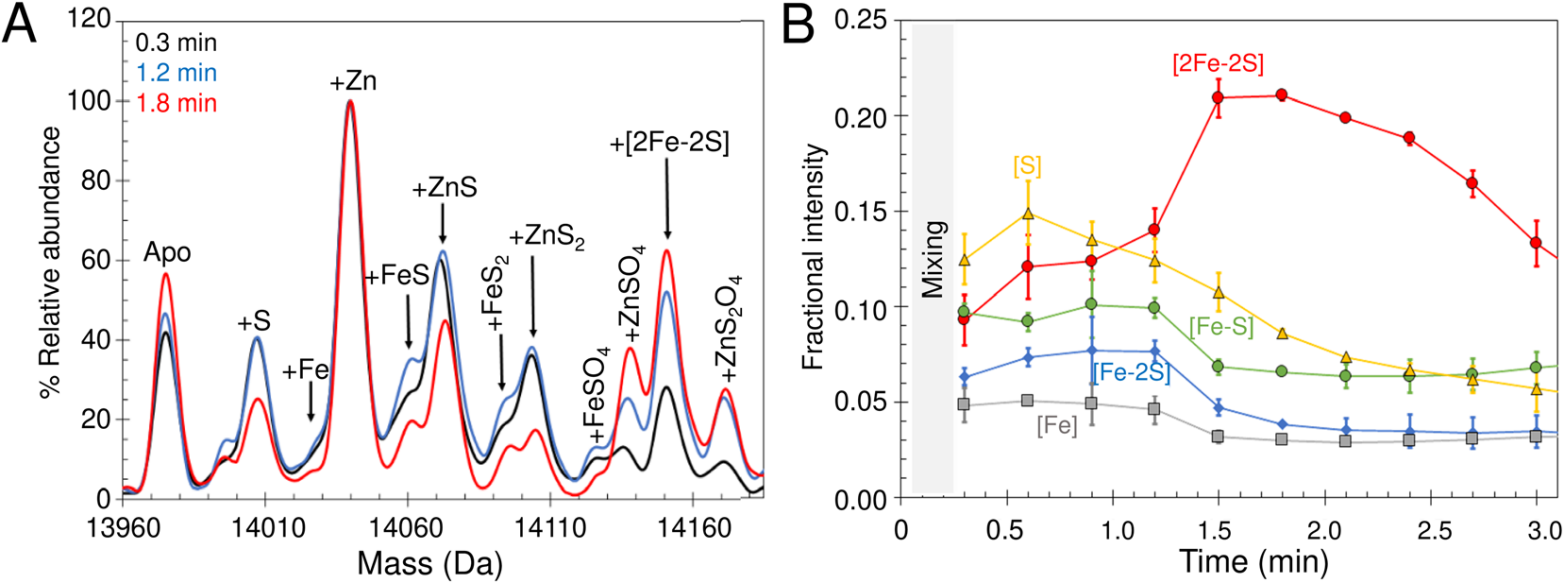
Non IscS-mediated formation of [2Fe–2S] cluster on IscU reveals assembly intermediates. (A) Deconvoluted mass spectrum of apo-IscU under conditions of spontaneous self-assembly. IscU was pre-treated for ∼50 s with a 54-fold excess of DTT-S generated by dissolving S^0^ in 200 mM ammonium formate buffer containing DTT (final ratio of S^0^ to DTT, 1 : 1.6) and then, immediately before infusion, the IscU sample was treated with a ∼18-fold excess (180 μM) of Fe^2+^ and mixed. Data were acquired from ∼20 s post iron addition. Major species are indicated. (B) Temporal behaviour of Fe–S cluster intermediates suggests [S] precedes the appearance of [Fe–S] and [Fe–S_2_], which in turn precedes the appearance of the [2Fe–2S] cluster. The gradual loss of [2Fe–2S] IscU beyond 2 min, as noted above, is consistent with the increased lability of the cluster in ammonium formate.

Initially, volatile buffer compositions and protein concentration that supported Fe–S cluster reconstitution were established and then optimized. Briefly, catalytic quantities of IscS were used initially (IscS : IscU ratio of 1 : 50), with apo-IscU in ammonium formate. Under these conditions cluster formation was partially inhibited compared to that observed in a typical Tris-buffered reconstitution, as monitored by absorbance/CD spectroscopy (Fig. 7A and 8A).^70^ Volatile buffers containing acetate were found to inhibit Fe–S cluster assembly, consistent with acetate inhibition of other PLP dependent enzymes;^71^ we note Lin *et al.* reported a 4-fold decrease in the desulfurase activity of IscS exchanged into ammonium acetate.^52^

Because only catalytic amounts of IscS were used in reconstitution reactions, and the added difficulties in resolving small increases in mass on large protein complexes (such as IscSU), native MS measurement conditions were optimised to favour the ionisation of free IscU, over the IscSU complex. At the start of the reconstitution, peaks corresponding to apo-IscU (13 976 Da) and Zn^2+^–IscU (14 039 Da) were observed (Fig. 7B, Table 1), along with various adducts. Over the course of the reconstitution (25 min), peaks due to apo- and Zn^2+^–IscU were significantly reduced and a mass gain of +175 Da (relative to apo-IscU) was observed, which corresponds to a [2Fe–2S] cluster (Fig. 7B and C). The charge state distribution observed for the product (Fig. 7C) was very similar to that observed for Zn^2+^–IscU, indicating that the cluster stabilises the folded form of the protein. Measurements over longer time periods revealed the gradual loss of the cluster-bound form of IscU, suggesting significant lability of the cluster.^72^

Reconstitution experiments were repeated as above but with ^57^Fe in place of natural abundance iron (^56^Fe), or ^34^S-cysteine as a source of sulfur in place of natural abundance (^32^S) cysteine.^51,54^ Cluster assembly in both cases was slower (Fig. S8†), possibly due to kinetic isotope effects,^73,74^ but generated the product [2Fe–2S] cluster with mass shifts, relative to the natural abundance spectrum, of +2 Da or +4 Da, respectively (Fig. 7D). The data demonstrate the incorporation of two irons and two sulfides into the nascent cluster. The time-resolved data did not reveal any clear intermediates of cluster formation, though the prevalence of sodium adducts of IscU tended to obscure regions of the spectrum where Fe/S complexes might be observed. Furthermore, under the conditions employed here, dimeric [4Fe–4S] IscU^33^ was not observed, even after prolonged incubation.

Thus, native MS provided unambiguous evidence of the incorporation of a [2Fe–2S] cluster into IscU, apparently without accumulating intermediates, resulting in a shift of the protein's conformational equilibrium towards the structured form. The cluster is labile and no evidence of reductive coupling of two [2Fe–2S] clusters to form a [4Fe–4S] cluster was observed.

### Intermediates of [2Fe–2S] cluster assembly on IscU with an IscS mimic

The lack of cluster intermediate species in the above reconstitution experiments may be a consequence of the apparent poor ionisation efficiency of apo-IscU and adducts from a complex reconstitution solution. Thus, we sought to simplify the reconstitution. Iron-sulfur clusters spontaneously assemble *in vitro* under anaerobic conditions if inorganic S^2−^ and Fe^2+^ ions are combined with an apo-form of an Fe–S protein in the presence of a thiol based reductant. Elemental sulfur (S^0^) was dissolved in ammonium formate buffer containing DTT to generate a low molecular weight IscS mimic (DTT-S) compatible with native mass spectrometry. IscU was pre-treated with excess DTT-S and then, immediately before infusion, with excess Fe^2+^. Peaks consistent with the presence of S, [Fe–S], [Fe–S_2_] and [2Fe–2S] adducts appeared during the reaction time course (Fig. 8A). The temporal behaviour indicated that S precedes the appearance of [Fe–S] and [Fe–S_2_], which in turn precedes the appearance of the [2Fe–2S] cluster (Fig. 8B). The gradual loss of [2Fe–2S] IscU beyond 2 min, as noted above, is consistent with the increased lability of the cluster in ammonium formate. These results give us a clear temporal sequence of the species that appear first during the reaction. The reciprocal reaction, in which Fe^2+^ was added first, gave a constant low level of the same IscU intermediates, with only [2Fe–2S]–IscU increasing during the reaction time course (not shown).

### Intermediates of [2Fe–2S] cluster assembly on IscU probed by discontinuous assay

**Fig. 9:**
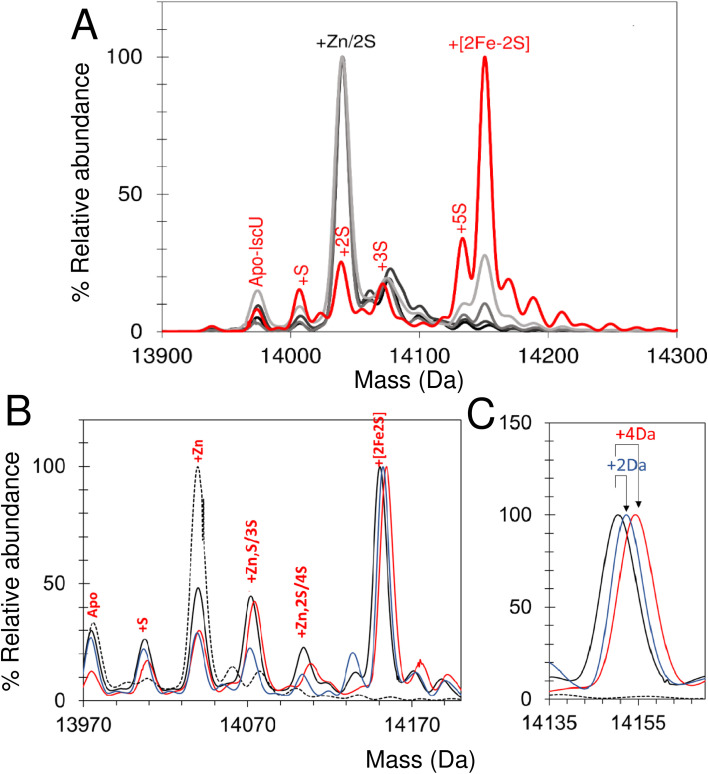
Assembly of [2Fe–2S] IscU monitored by discontinuous assay. (A) Reconstitution of apo-IscU in 50 mM Tris, 150 mM NaCl, pH 8 for increasing time periods and exchange into 250 mM ammonium acetate, pH 8 prior to native mass spectrometric measurement. Starting and end (15 min) spectra are in black and red, respectively. Intervening spectra are in increasingly light grey (1–5 min). (B) Deconvoluted mass spectra of IscU reconstituted in Tris buffer for 15 min before buffer exchange into 200 mM ammonium formate, pH 8.0. DTT-reduced apo-IscU (black dotted line), ^56^Fe and ^32^S-cysteine reconstitution (black), ^57^Fe reconstitution (blue), and ^34^S reconstitution (red). (C) Expanded view of the [2Fe–2S] cluster region.

In an alternative approach, IscU (<0.1 Zn^2+^ per IscU) reconstitution reactions in Tris buffer were allowed to proceed for increasing time periods, prior to buffer exchange into ammonium acetate. This had the effect of quenching the reconstitution reaction and removing low molecular weight products and reactants (that could potentially interfere with IscU ionisation). Samples were then immediately infused into the ESI-MS to capture newly formed intermediates and products associated with IscU. Fig. 9A shows the deconvoluted spectrum of IscU at specific time points during the first 15 min of the cluster reconstitution, which, based on absorbance data (Fig. 7), is close to completion.

After 2.5 min, the peak associated with a [2Fe–2S] cluster bound form of IscU was clearly observed. Adducts of apo-IscU containing Zn^2+^ or S (multiples of +32 Da, as previously reported^75^) were also observed. After 15 min, [2Fe–2S]–IscU at 14 150 Da was the most abundant species. As observed for continuous assay experiments above, the peak due to Zn^2+^–IscU (in this sample, <10% of total IscU) was significantly reduced during the reaction. Because these samples were buffer-exchanged before injection into the instrument, it was possible to determine whether the decrease in the abundance of the Zn^2+^–IscU peak was due to dissociation of Zn^2+^ from IscU. ICP-MS analysis of identical samples showed that, after 15 min reconstitution, very little Zn^2+^ (<0.01 Zn^2+^ per IscU) was protein-bound, thus demonstrating that Zn^2+^ is indeed displaced during the cluster assembly reaction.^52^

Isotopic substitution using ^34^S-cysteine as sulfur source assisted in the assignment of intermediate species (Fig. 9B and C and S9†). All IscU adducts were shifted to a heavier mass in the ^34^S reaction. The single S adduct was shifted by the expected +2 Da to +34 Da. The Zn^2+^–IscU peak, which was present at the start and significantly decreased as the reaction proceeded, would not be expected to shift, unless it overlapped with a peak due to an adduct of 2S. A shift was observed, but only of +2 Da, indicating that the peak arises from overlapping peaks due to Zn^2+^/2S adducts of IscU. The shifts observed for high mass adduct peaks at +96 Da and + 128 Da indicated that they, too, result from overlapping Zn^2+^/S species (Fig. S9†). We note that persulfide exchange could lead to the accumulation of S_*n*_, where *n* ≥ 2.

An equivalent isotope substitution experiment with ^57^Fe revealed no shifts in any of the apo-IscU adducts over the course of the reconstitution (Fig. 9B). Thus, in this discontinuous assay, the only stable iron species detected in complex with IscU was that incorporated into the product [2Fe–2S] cluster. We note that labile Fe^2+^ ions may be lost from IscU during the buffer exchange process, and that apparent Fe^2+^-binding affinity is lower in the ESI experiment.

### Persulfide forms of IscU are competent for cluster assembly

The above experiments revealed persulfide forms of IscU are readily formed. To investigate whether such species can accumulate in the absence of iron, assays were conducted (in ammonium formate) with varying concentrations of cysteine and IscS. Cysteine at 5-fold (as in reconstitution reactions), and at 20-fold excess over IscU, was used, with catalytic amounts of IscS (IscU : IscS 50 : 1) (Fig. S10A†). In additional experiments a higher concentration of IscS (IscU : IscS 2 : 1) was also employed to increase the availability of sulfur. In all cases, S adducts similar to those observed above (and confirmed using ^34^S-cysteine, Fig. S10B†) were observed, demonstrating that S transfer from IscS to IscU can also occur in the absence of iron. At higher cysteine concentration, adducts of IscU with one and two cysteines were also detected, Fig. S10A.† Also noteworthy was that, in the presence of IscS/cysteine, apo-IscU was the highest abundance peak (rather than Zn^2+^–IscU), indicating the displacement of Zn^2+^ from IscU in the presence of IscS/cysteine, and/or improved ionisation efficiency of apo-IscU under these conditions. A reconstitution reaction with the higher concentration of IscS was also carried out (Fig. S11†), confirming that [2Fe–2S] assembly on IscU occurs under these conditions.

The above experiments indicate that transfer of S^0^ from IscS to IscU can readily occur in the absence of iron. Previous reports suggested that such species are off pathway products that are unable to facilitate [2Fe–2S] assembly.^56^ To investigate this further, the cluster assembly process, followed by UV-visible absorbance, was broken down into two steps: IscS-mediated addition of S^0^, and subsequently addition of iron and reductant (Fig. S12†). Cysteine (5-fold excess) and catalytic amounts of IscS (IscU : IscS 50 : 1) were added to IscU under aerobic conditions, and unbound small molecules removed by gel filtration, resulting in persulfide-containing IscU (Fig. S12A†). Under anaerobic conditions, Fe^2+^ alone (Fig S12B†) and in combination with reductant (DTT) was added to persulfide-IscU. The inclusion of reductant resulted in the rapid (within 5 min) formation of [2Fe–2S] (Fig. S12C†). Alternative reductants GSH and cysteine were also employed, in both cases resulting in cluster formation but over a longer period (Fig. S12D and E†). In the case of cysteine, further reaction was observed, presumably because of turnover by IscS.

Overall, the data above demonstrate that S adducts readily accumulate on IscU both in the presence and absence of Fe, and are available and fully competent for [2Fe–2S] cluster assembly (Fig. S12F†).

### Effect of Zn^2+^ on [2Fe–2S] cluster assembly on IscU

**Fig. 10:**
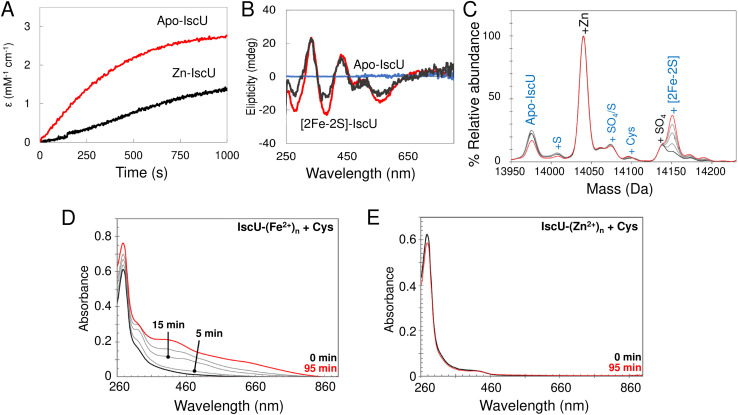
The effect of Zn^2+^ on the reconstitution of [2Fe–2S] IscU. (A) and (B) Reconstitution of apo-IscU (red) and Zn^2+^–IscU (grey) in 50 mM Tris, 150 mM NaCl, pH 8.0, monitored by absorbance (A) or CD (B) spectroscopies. (C) Zn^2+^–IscU (10 μM) in 200 mM ammonium formate buffer, pH 8.0 was reconstituted using 20 μM Fe^2+^, 50 μM cysteine, 2 mM DTT and 0.2 μM IscS. The reaction was directly infused into the MS once the IscS was added. The starting spectrum is in black and the end spectrum (at 25 min) is in red (intervening spectra in grey). Adducts of apo-IscU and Zn–IscU are described in blue and black, respectively. (D) IscU pre-loaded with excess Fe^2+^ ions retained 0.68 (±0.06) Fe^2+^/IscU post-desalting, and supported Fe–S cluster assembly in the presence of 5 μM IscS and 2 mM l-cysteine. (E) IscU pre-loaded with excess Zn^2+^ ions, then Fe^2+^ ions, retained 1.65 (±0.35) Zn^2+^/IscU, but contained no detectable Fe^2+^ ions post desalting. The addition of 5 μM IscS and 2 mM l-cysteine failed to support Fe–S cluster assembly, consistent with the lack of Fe^2+^ ions.

In order to assess the effect of bound Zn^2+^ on cluster assembly, reconstitution experiments as described above (*i.e.* with IscU in the presence of catalytic quantities of IscS) were carried out with fully Zn^2+^-loaded IscU. These resulted in a significantly reduced rate of assembly, but still yielded [2Fe–2S] IscU (Fig. 10A and B). This is consistent with some previous reports,^76,77^ but not all.^34^ Native mass spectra revealed formation of a [2Fe–2S] cluster bound to IscU without Zn^2+^ (Fig. 10C), indicating that Zn^2+^ was displaced during cluster assembly, as was also reported in a recent native MS study.^52^

The displacement of Zn^2+^ may be of physiological importance. To probe this further, cluster reconstitution experiments were performed, separately, with apo-IscU and fully loaded Zn^2+^–IscU in regular reconstitution Tris buffer. First, a 30-fold excess of Fe^2+^ was added anaerobically to each. Unbound excess Fe^2+^ was subsequently removed by gel filtration. Catalytic IscS and cysteine (3 mM) was then added and cluster assembly followed by UV-visible absorbance. For the sample originally containing apo-IscU, the protein was found to contain ∼0.7 Fe^2+^ per protein following gel filtration, and clear evidence of cluster assembly was apparent at 20 min (Fig. 10D). For the sample originally containing Zn^2+^–IscU, no bound Fe^2+^ was detected following gel filtration and cluster assembly was thus not observed (Fig. 10E).

These data demonstrate that, while Zn^2+^ inhibits cluster assembly on IscU, it does not abolish it; this is because the cation can be displaced from IscU. However, a 30-fold excess of Fe^2+^ had no effect on Zn^2+^-binding, indicating that it is the transfer of S^0^ from IscS that promotes displacement. Furthermore, the data indicate that Fe^2+^ binds at the same (or similar) site as that occupied by Zn^2+^.

## Discussion

Iron–sulfur clusters spontaneously assembly *in vitro* under anaerobic conditions if S^2−^ and Fe^2+/3+^ ions are combined with apo-protein in the presence of a thiol-based reductant. In contrast, living organisms coordinate the availability of potentially toxic iron and sulfide ions *via* a multiprotein machinery that *de novo* synthesizes Fe–S clusters on a dedicated scaffold protein. Crystal structures of the sulfide-generating cysteine desulfurase, IscS, alone and in complex with apo- and [2Fe–2S]-forms of the scaffold protein IscU, are available.^11,14,17^ These provide key insight into the mechanism of inter-molecular sulfur transfer and protein–protein interactions that facilitate the successful formation of [2Fe–2S]–IscU. However, despite the apparent simplicity, the precise mechanistic sequence of events of Fe–S cluster assembly remains elusive.

The application of native MS has provided extraordinary mechanistic insight into Fe–S cluster proteins,^45,46,48^ but major challenges in using this technique to investigate intermediates in the Fe–S assembly process exist, including efficient ionisation of large IscS–IscU complexes (∼105 to 120 kDa), and difficulties in achieving sufficient resolution to distinguish the addition of single atoms/ions (*e.g.* S^32^, S^34^, Fe^56^, Fe^57^) to large complexes. For this reason, we optimised the experiments to enable detection of IscU and Fe/S adduct species.

While the work described here was underway, Lin *et al.*^52^ reported their native MS study of IscSU-mediated Fe–S assembly, in which they identified potential Fe–S cluster intermediates and products associated with IscU. Here we have implemented a complementary set of native MS experiments. Our data are broadly consistent with the previous study, but offer significant further insights into the enigmatic process of Fe–S cluster assembly through the use of ^34^S and ^57^Fe isotopes to investigate S- and Fe-binding/transfer, and the behaviour of Zn^2+^–IscU.

### Conformational flexibility of IscU and Zn^2+^- and Fe^2+^-binding

Various biophysical approaches, including NMR spectroscopy, have shown IscU to be a dynamic protein that readily interconverts between two different conformations on a second time scale *in vitro*:^34,66,77,78^ one conformation (S-form) is largely structured, the other (D-form) is dynamically disordered. We note that Sato *et al.* have recently demonstrated that the interconversion between S- and D-forms of IscU is a fundamental requirement for the assembly and transfer of Fe–S clusters *in vivo*.^79^ The presence of Zn^2+^ ions, or Fe^2+^ ions (and indeed a [2Fe–2S] cluster), shifted the equilibrium between D- and S-forms in favour of the S-form (Fig. S13†), consistent with previous solution studies.^20,38,53,78,80^ The presence of Zn^2+^ ions also reduces the tendency of apo-IscU to dimerise and stabilises monomeric IscU in the S-form. Li *et al.* recently demonstrated that zinc inhibits IscU/IscA-mediated Fe–S assembly and transfer, rather than disrupting already assembled clusters,^39^ and data presented here are consistent with this (Fig. 10). We also note that IscU is a substrate for degradation by ClpXP and FtsH proteases, and it is possible that Zn^2+^–IscU may be more resistant to the effects of these proteases than apo-IscU.^81^

*E. coli* IscU is isolated containing significant amounts of Zn^2+^. Here we showed that Zn^2+^ is displaced from IscU during cluster assembly, and that this is dependent on the presence of IscS and Cys, where S^0^/S^2−^ is generated, consistent with previous reports.^52^ This occurred even in the absence of Fe^2+^ (where Fe–S assembly could not occur). It is possible that transfer of the persulfide formed on IscS–Cys321 during the process of cluster assembly triggers the displacement of Zn^2+^, perhaps by altering the Zn^2+^ binding environment of IscU to generate a less optimal, lower affinity site.

In contrast to zinc, the ‘free’ iron pool is 10–30 μM under aerobic conditions, rising several fold under anaerobic conditions.^82,83^ We have determined the *K*_d_ of Fe^2+^ for *E. coli* IscU to be ∼3.5 μM, consistent with literature values for IscU homologues.^59,67–69^ Taken together, these values suggest that Fe^2+^–IscU should exist as a major species *in vivo* under aerobic and anaerobic conditions, although we are unaware of any successful retrieval of Fe^2+^–IscU from an *in vivo* source.

Interestingly, we found that the *K*_d_ of Fe^2+^-binding under the conditions of the native MS experiment was significantly higher (∼73 μM), accounting for why high concentrations of Fe^2+^ were necessary to observe Fe^2+^-bound forms by MS.^52^ Thus, it appears that something occurs during ionisation/transfer to the gas phase that affects the affinity of IscU for Fe^2+^. This is not typical of protein–metallocofactor interactions measured by native MS, and does not appear to be general for divalent metal ion binding, because Zn^2+^-binding was not affected. One possibility is that the Fe^2+^-binding site features a water as a coordinating ligand, which may be lost during ionisation/transfer to the gas phase, where the solvent is removed. A metal binding site lacking a coordinating ligand would be expected to exhibit lower affinity, as observed.

### IscS–IscU interactions

Native MS experiments revealed that both apo- and Zn^2+^–IscU can from a largely structured complex with IscS, with no significant difference in stability.^52^ As the concentration of IscU was increased, we observed the sequential formation of (IscS)_2_IscU and (IscS)_2_(IscU)_2_. The former maximised at IscU/IscS ≈ 1.0, with the concomitant loss of un-complexed dimeric IscS, whereas the (IscS)_2_(IscU)_2_ complex was evident at IscU/IscS ≥ 1.0. The data indicate that dimeric IscS has two equivalent, independent (non-cooperative) binding sites for IscU (*K*_d_ ∼ 3 μM). We note that, *in vivo*, IscS and IscU concentrations are approximately equal (∼15 μM each^84^), suggesting a mix of (IscU)(IscS)_2_ and (IscU)_2_(IscS)_2_, with almost no free (IscS)_2_.

### Mechanism of [2Fe–2S] cluster assembly

The biological assembly of Fe–S clusters requires a multiprotein machinery that *de novo* synthesizes a Fe–S cluster on a dedicated scaffold protein (*e.g.* IscU) prior to transfer to client proteins. *Escherichia coli* possesses two distinct systems, the ISC (*iscRSUA-hscBA-fdx-iscX*) and SUF (*sufABCDSE*) systems, which are utilised during standard growth or conditions of oxidative stress/iron limitation, respectively. In a Δ*suf* background, Tanaka *et al.* found that *iscSU* represents the minimal requirement for biosynthesis under anaerobic conditions.^85^ The human pathogen, *H. pylori*, a micro-aerobe, naturally contains a minimalist Fe–S system (*nifSU*) that is essential for viability and is functionally exchangeable for *E. coli* Fe–S assembly systems.^86^ Minimalist Fe–S assembly systems likely evolved before their more complex counterparts, *e.g.* ISC,^87^ suggesting that Fe acquisition is likely an inherent property of the IscSU complex or its constituent parts, at least under anaerobic conditions.

Our reconstitutions, conducted with catalytic quantities of IscS, were monitored by time dependent native MS, using both continuous and discontinuous assays. As noted above, the intensity of the Zn^2+^–IscU peak decreased with a concomitant increase in the amount of [2Fe–2S]^2+^ IscU. No significant Fe–S intermediates were observed on IscU. A range of IscU–S adducts, but no Fe^2+^-only IscU species, were detected: in all cases, apo-IscU was observed to transition directly to [2Fe–2S]–IscU during the reaction time course. To gain access to Fe–S cluster assembly intermediates, it was therefore necessary to adopt an approach in which S^0^ transfer was not optimised, achieved here using a low molecular weight IscS mimic. Peaks consistent with the presence of IscU-bound [S], [Fe–S], [Fe–S_2_] and [2Fe–2S] were observed during the reaction time course. The temporal behaviour indicates that [S] precedes the appearance of [Fe–S] and [Fe–S_2_], which in turn precedes the appearance of the [2Fe–2S] cluster.

Overall, the data indicate that the IscS-catalysed assembly reaction on IscU is largely concerted, such that intermediate species do not readily accumulate to detectable levels. Intermediates were observed in the absence of IscS, where an IscS-mimic was employed, probably because the reaction without IscS was sub-optimal, allowing for some accumulation of intermediates.

**Fig. 11:**
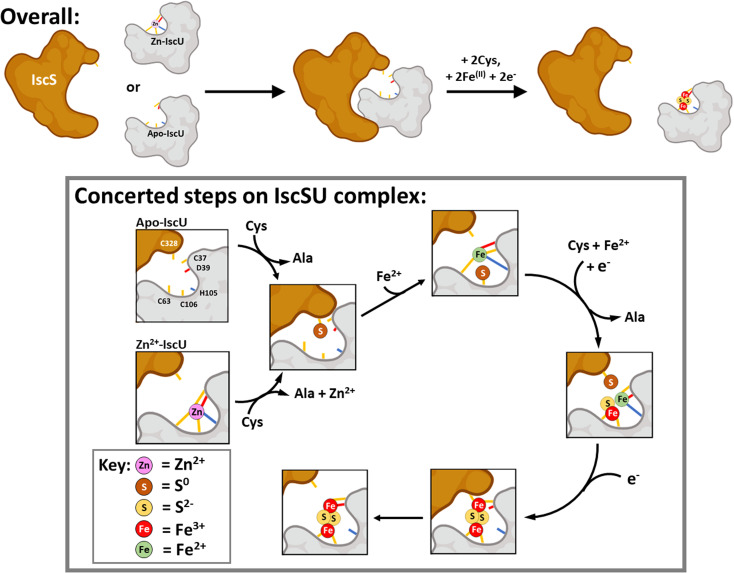
Scheme describing the proposed mechanism of assembly of [2Fe–2S] on IscU in complex with IscS. The difficulty in detecting intermediates of cluster assembly points to a conserved mechanism,^17^ in which assembly of a [2Fe–2S] goes to completion in preference to the initiation of cluster formation on another IscU molecule, with the effect that assembly intermediates don't accumulate. Details of S- and Fe-binding/transfer are speculative, based on data presented here and previously. The Cys residue of IscU to which S^0^ is transferred from IscS is not known. Evidence for the involvement of the residues equivalent to Cys37 and Cys106 in the human homologue IscU is available.^53,88,89^ Inspection of the structure of IscS/IscU suggests that Cys37 is the most likely residue for initial transfer, prior to arrival at Cys106.

Based on our observations and previous work, a working model for Fe–S assembly (Fig. 11) starts with apo-IscU (or Zn^2+^–IscU) docking to a vacant site on the IscS dimer. Although, anchored by intra-molecular salt bridges and hydrogen bonds, IscU remains dynamic, ensuring access to the Fe–S assembly site, as well as the inter-molecular catalytic centre. Hence, all subsequent steps in the assembly processes most likely take place on the IscU–IscS complex. Upon binding, and in the presence of substrate cysteine, IscS donates two successive sulfane (S^0^) atoms to IscU^17^*via* a persulfide generated by IscS on Cys321 and involving non-redox transfer of the S^0^ (*trans*-persulfidation).^19^

Our results suggest that IscS-mediated addition of S^0^ to IscU creates a site that has lower affinity for Zn^2+^, permitting displacement by Fe^2+^ ions at the assembly site, probably involving persulfide-ligated Fe^2+^. A crystal structure of the *Archaeoglobus fulgidus* IscS–IscU^D35A^ complex containing a partly oxidised [2Fe–2S] intermediate with a unique μ_2_–η3 sulfido ligation indicates that persulfide coordination of iron at the cluster site is possible.^17^

Overall, four electrons are required to reduce two S^0^ to S^2−^ ions for the synthesis of one [2Fe–2S]^2+^ cluster. The oxidation of the incoming Fe^2+^ ions to Fe^3+^ ions may provide two of the four required electrons, but we note that Fe^2+^ in the absence of IscS was not efficient in reducing S^0^ species on IscU. Alternatively, or additionally, electrons are supplied by the ferredoxin encoded by *fdx* (note that Fdx is not essential *in vivo* under anaerobic conditions,^85^ and that electrons can be alternatively supplied, *e.g.* by Cys and/or GSH). The exact sequence of events, including the order in which Fe^2+^-binding/sulfur transfer to IscU occurs, and how Fdx binding on IscS facilitates electron transfer to S^0^ is not clear. The time-resolved data reported here suggest that S^0^ is first transferred and one Fe^2+^ then binds, followed by a second S^0^/Fe^2+^ to generate the product cluster.

Previous structural studies of *A. fulgidus* IscS–IscU^D35A^ revealed a cluster coordination consisting of the three Cys residues of IscU along with Cys321 of IscS.^17^ Molecular dynamic simulations suggested the dissociation of the complex involves the recruitment of IscU Asp35 as a cluster ligand, displacing Cys33, which in turn displaces IscS–Cys321, leading to the dissociation of [2Fe–2S]^2+^–IscU from the IscS–IscU complex.

Overall, our observations are broadly consistent with those of Lin *et al.*^52^ in that Fe–S assembly can be initiated *in vitro* from Fe^2+^ or S^0^ bound forms of IscU. However, our data point to IscSU mechanism of Fe–S cluster assembly that is concerted, in which either iron or sulfur can bind first. In support of this are the following observations: (1) cluster Fe/S intermediate species are not readily observed on ‘free’ IscU during IscS-mediated [2Fe–2S] assembly, indicating that once assembly begins on the IscSU complex, it goes to completion before cluster assembly is initiated on another IscSU complex; (2) sulfur adducts are readily observed on ‘free’ IscU both under cluster assembly conditions, and when Fe^2+^ is absent (Fig. S11†), as reported by others,^19,75^ consistent with facile transfer from IscS to IscU. Transferred sulfur can subsequently support cluster assembly. Thus, Fe^2+^-binding is not a pre-requisite for S^0^ transfer; (3) IscU pre-loaded with Fe^2+^ can also support cluster assembly when IscS/cysteine are introduced.

While the order of Fe^2+^-binding and sulfur transfer does not appear to matter *in vitro*, it seems likely that the process of cluster assembly on IscU is controlled by sulfur availability, such that sulfur transfer is the key step. The presence of Zn^2+^ on IscU entirely inhibits Fe^2+^-binding and prevents the formation of dimeric IscU. However, IscS/cysteine promotes the removal of Zn^2+^ from Zn^2+^–IscU,^52^ facilitating Fe^2+^-binding and cluster assembly. This must involve/require S^0^ transfer and points to sulfur transfer as the key initiating step of assembly. This is consistent with the regulation of Isc Fe–S cluster assembly by CyaY at the level of S^0^ generation/transfer.^81,90^ The supply of IscS-generated S^0^ in the cell is carefully regulated, by proteins such as CyaY and IscX,^22,26^ which tune the activity of IscS through competition binding. Tight regulation of S^0^ generation/transfer is consistent with this being the key committed step, rather than reversible Fe^2+^-binding (where Fe^2+^ is supplied by an iron donor, or more likely from the chelatable iron pool), in cluster assembly.

### Concluding remarks

We have applied native mass spectrometry alongside more traditional methods to gain insight into key aspects of the properties and behaviour of the Fe–S cluster scaffold protein IscU, and in particular the chemistry taking place during IscU-mediated [2Fe–2S] cluster assembly. Two conformations of IscU were readily detected, corresponding to the previously characterised S- and D-states. IscU was isolated in a Zn^2+^-bound form that existed exclusively in the S-state, but Zn^2+^ could be removed using a high affinity chelator, or through the assembly of a [2Fe–2S] cluster on IscU. Cluster assembly was followed using time-dependent mass spectrometry, with a lack of observable Fe–S intermediates on ‘free’ IscU suggesting a concerted reaction. Although Fe–S cluster assembly can be initiated *in vitro* from Fe^2+^- or S^0^-bound forms of IscU, when Zn^2+^ is bound to IscU, sulfur transfer is required to initiate cluster assembly through the displacement of Zn^2+^, which enables Fe^2+^ to bind at the assembly site. The proposed sulfur-initiated assembly process is consistent with the allosteric regulation of IscS sulfur production/transfer by CyaY and IscX.

## Data availability

Data supporting the conclusions of this study are available in the main paper with additional experimental data given in the ESI.†

## Author contributions

JCC, AP and NLB conceived the study and contributed to experimental design. SPB, JCC and RP contributed to protein production and purification. SPB and JCC carried out the Fe–S cluster assembly experimental work. SPB, JCC and NLB analysed the data. JCC and NLB wrote the paper with assistance from RP and AP. All authors have approved the final version of the manuscript.

## Conflicts of interest

The authors have no conflicts to declare.

## Supplementary Material

SC-014-D2SC04169C-s001
