# Supplementary material for: Native mass spectrometric studies of IscSU reveal a concerted, sulfur-initiated mechanism of iron–sulfur cluster assembly
Source: Chem Sci. 2022 Nov 15;14(1):78–95. doi: 10.1039/d2sc04169c (PMC9769115; doi:10.1039/d2sc04169c)
Supplement: SC-014-D2SC04169C-s001 [file SC-014-D2SC04169C-s001.pdf]

Electronic Supplementary Material (ESI) for Chemical Science.
This journal is © The Royal Society of Chemistry 2022

Supplementary Information

Native mass spectrometric studies of IscSU reveal a concerted, sulfur-initiated mechanism of iron-sulfur cluster assembly.

Sophie P. Bennett, Jason C. Crack, Rita Puglisi, Annalisa Pastore, Nick E. Le Brun

Supplementary Schemes

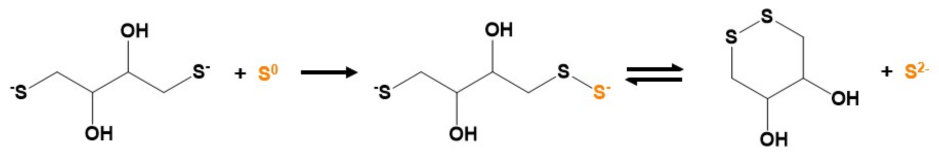

Scheme S1. The reaction of DTT with sulfane sulfur leading to persulfuration of DTT. The equilibrium arrows indicate the reversible oxidation of the persulfide to generate disulfide bonded DTT and sulfide (S^{2-}).

Supplementary Figures

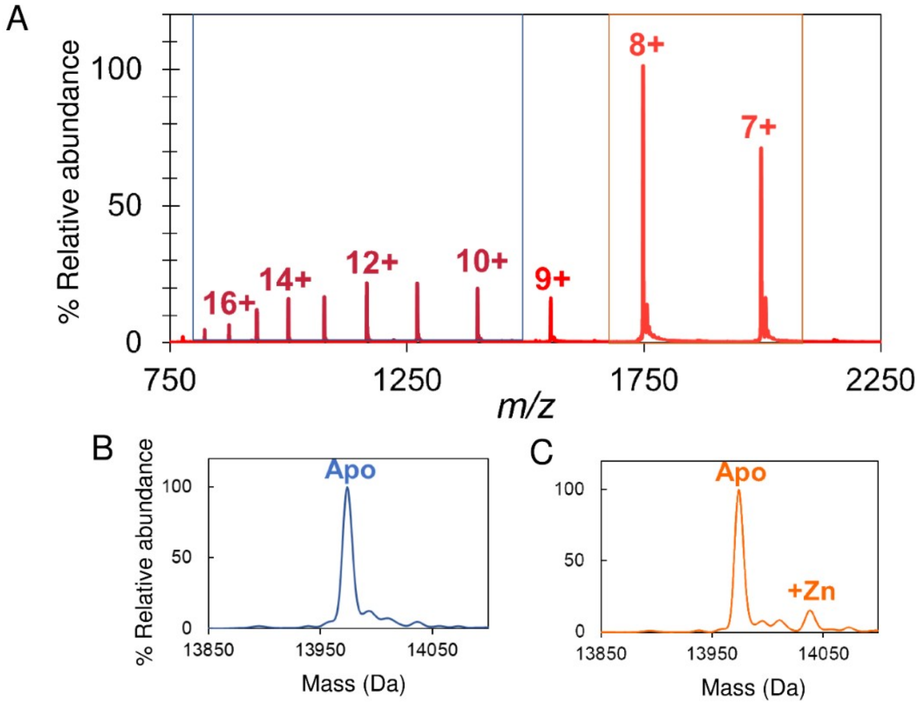

Figure S1. Native mass spectrometry analysis of the D and S states of IscU. (A) *m/z* spectrum of IscU following removal of Zn^{2+}. (B) and (C), Deconvolution of two separate *m/z* regions in the apo-IscU spectrum, representing apo-IscU in the 10-17+ charge states (boxed in blue in (A)) and a mixed apo and Zn associated IscU in the 7+ and 8+ charge states (boxed orange in (A)).

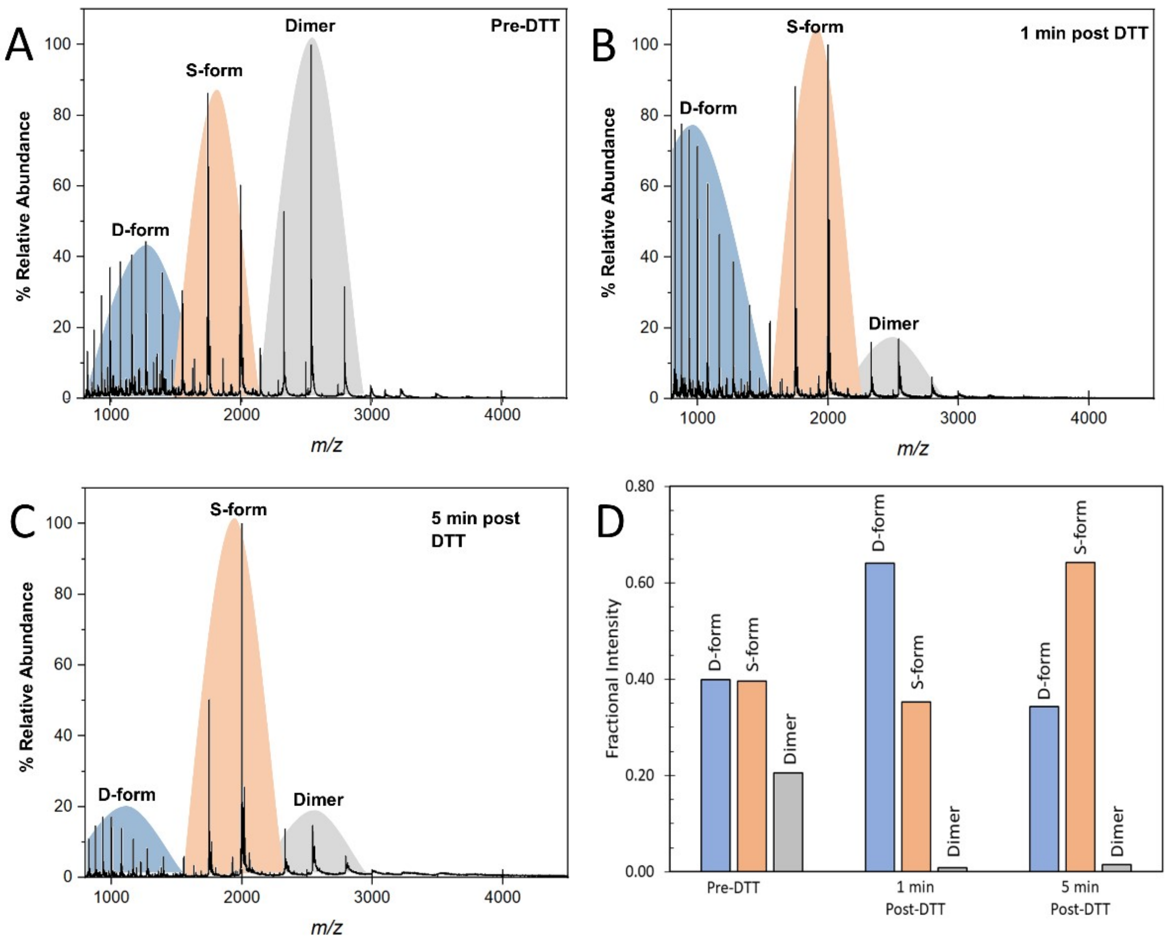

Figure S2. Reduction of oxidised IscU by DTT. (A) *m/z* spectrum of apo-IscU (~10 μM in 250 mM ammonium acetate pH 8) prior to the addition of DTT. The charge state distribution indicates the presence of D and S forms of monomeric IscU, and also dimeric IscU. (B) and (C) *m/z* spectra of apo-IscU following the addition of 7.6 mM DTT to apo-IscU after 1 and 5 min, respectively. (D) Fractional intensity of D, S and dimeric forms of IscU pre- and post-DTT treatment, as indicated.

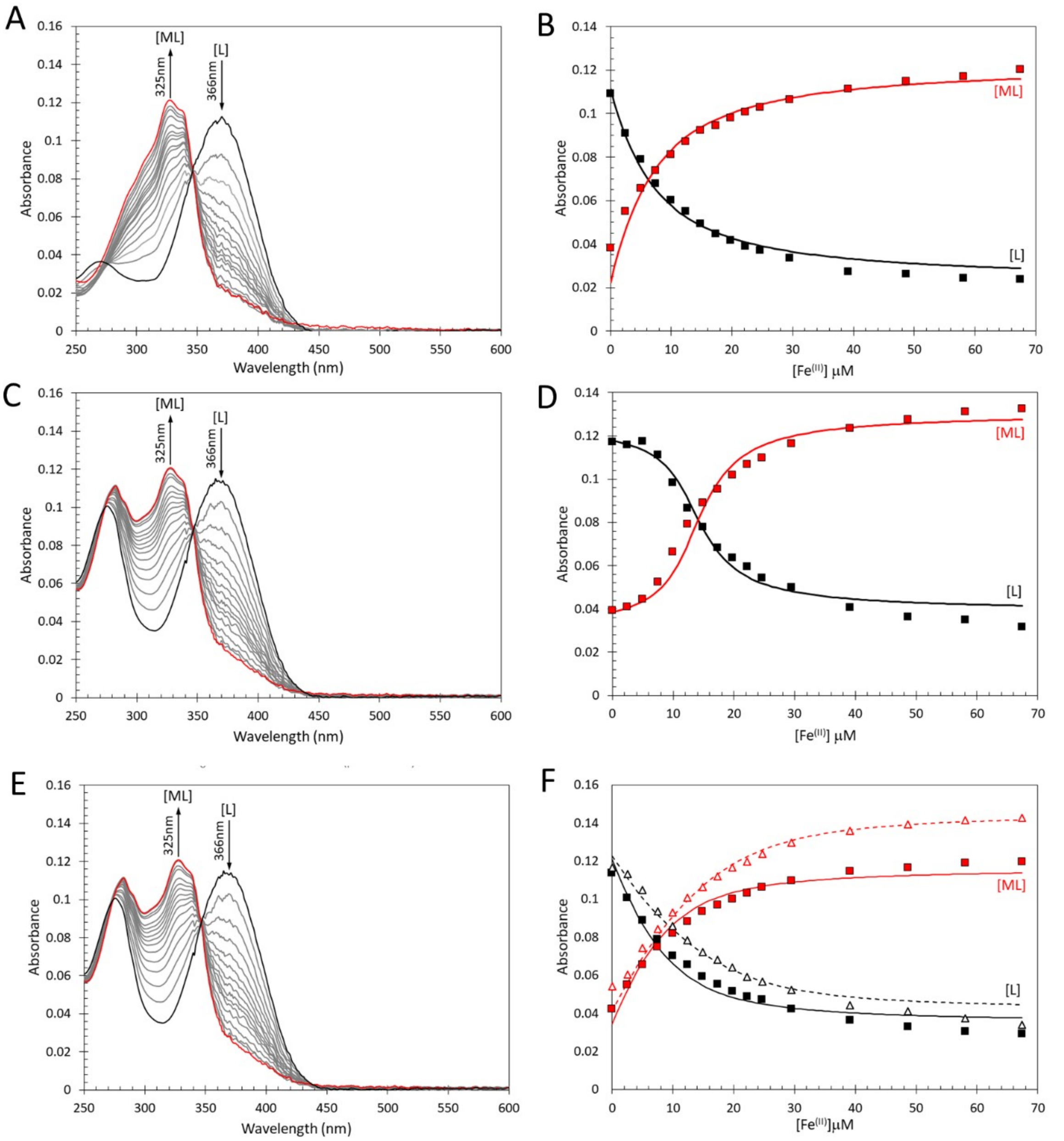

Figure S3. Effect of potentially competing formate on the affinity of Fe^{2+} for IscU and mag-fura-2. (A) Spectra showing Fe^{2+} binding to mag-fura-2 (5 μM) in 200 mM ammonium formate, pH 8.0. Arrows indicate direction of absorbance changes for apo- and Fe^{2+}-mag-fura-2, [L] and [M.L], respectively. (B) Plot of absorbance changes at 325 nm (red points) and 366 nm (black points) from (A) and fit (solid line, see Methods), yielding a K_d for Fe^{2+} binding to mag-fura-2 in the presence of ammonium formate of 2.0 ±0.1 μM. (C) and (D) as in (A) and (B) but in the presence of competing apo-IscU (5 μM) passed once down a desalting column (PD10 Cytiva) to remove DTPA. The sigmoidal response indicates the presence of a species with a higher affinity for Fe^{2+} than mag-fura-2. Fitting to a simple binding equation describing competition between mag-fura-2, apo-IscU and an unknown species indicated an apparent K_d of 0.27 ±0.04 μM and concentration of ~12 μM for the unknown species. Thus, ~2 molecules of the unknown species are associated with each IscU post desalting, which inhibits Fe^{2+}-binding. The unknown species was removed (or at least its effect on Fe^{2+} binding was removed) by a second pass down a desalting column, suggesting it arises from a low

molecular weight Fe^{2+}-binding species that is associated with apo-IscU. This is likely to be DPTA. Data from a repeat of the competition experiment following 'clean-up' of apo-IscU are shown in (E) and (F). In (F) data for Fe^{2+} binding to mag-fura-2 in competition with 5.0 μM (closed circles, solid lines) and 15.0 μM (open triangles, dashed lines) apo-IscU was fitted as above. These reveal an apparent K_d of 3.0 ±0.9 μM, similar to that measured in HEPES buffer (see Fig. 4) and consistent with literature reports[1, 2].

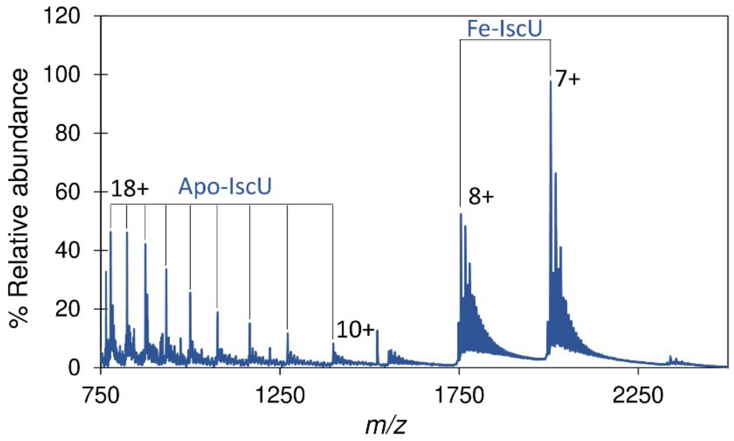

Figure S4. *m/z* spectra of IscU following addition of excess Fe^{2+}. The charge state distribution indicates the presence of two conformational forms, corresponding to apo-IscU (in the D state) and Fe^{2+}-IscU (in the S state). Thus Fe^{2+} binds to IscU with much lower affinity than does Zn^{2+}, but it has a similar effect on the conformation of IscU, stabilising the S state over D state.

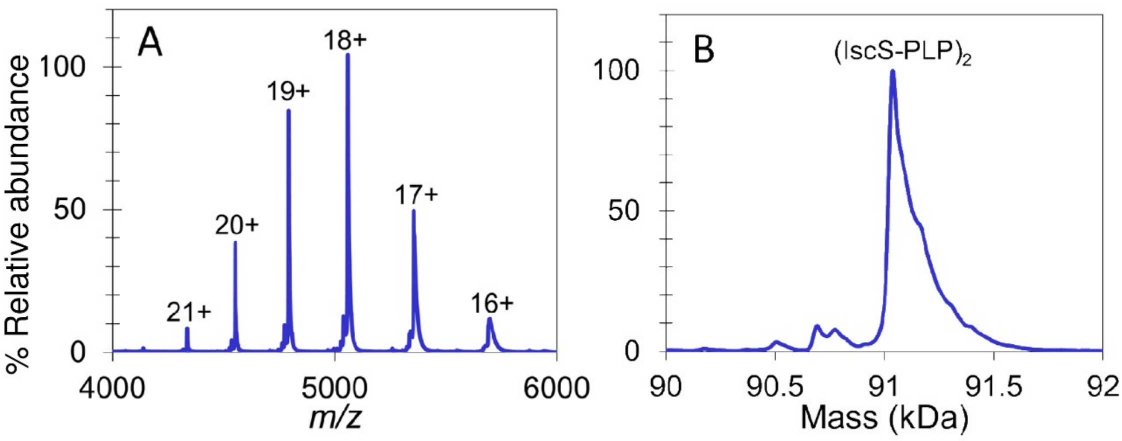

Figure S5. Native mass spectrometric measurement of IscS in 200 mM ammonium formate, pH 8.0. The acquired *m/z* spectrum (A) and deconvoluted to neutral mass spectrum (B). At the higher *m/z* values measured in (A), the resolution is lower, ~2500 for +18 charge state, compared to ~5500 for +8 charge state of IscU (shown in Fig. 2A), leading to a broad IscS peak (B).

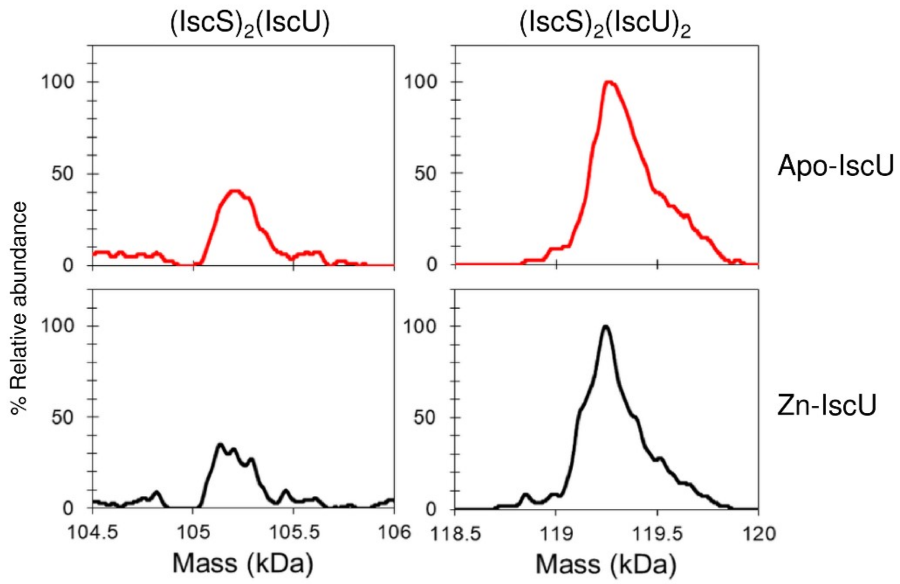

Figure S6. Association of IscS with apo-IscU or Zn-IscU investigated by native mass spectrometry. Deconvoluted mass spectra in the mass ranges corresponding to $(IscS)_2$IscU and $(IscS)_2(IscU)_2$ complexes. IscS (8 µM) was in 250 mM ammonium acetate, pH 8 with IscU (apo- or Zn-) at a ratio of 1:2.

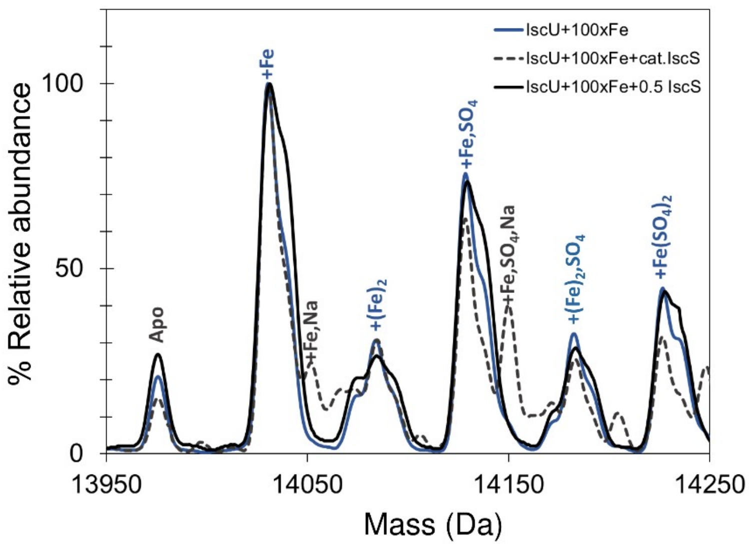

Figure S7. Fe^{2+}-binding to IscU in the presence of IscS. Incubation of IscU in 200 mM ammonium formate, with 1 mM DTT and a 100 fold excess ferrous ammonium sulfate and IscS as a catalytic amount and as a ratio of 2:1 IscU:IscS.

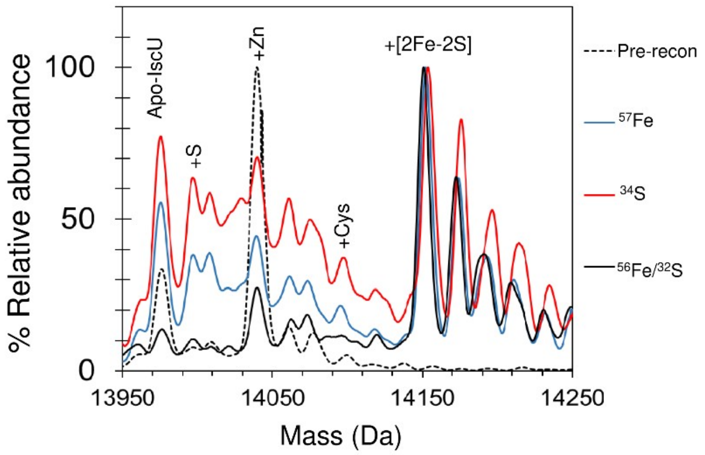

Figure S8 Reconstitution of apo-IscU after 25 min infusion into the MS using isotopes of Fe and S. Samples were prepared in 200 mM ammonium formate, pH 8.0 and reconstituted using the same method as in Fig. 7. Apo-IscU and 2 mM DTT (black dotted), ^{56}Fe and ^{32}S – cysteine reconstitution (back), ^{57}Fe reconstitution (blue), and ^{34}S reconstitution (red). The relative intensities of [2Fe-2S] and apo-IscU observed after 25 min indicate that the reaction is slower in the presence of either ^{57}Fe or ^{34}S, with a greater effect observed for ^{34}S. This could arise from kinetic isotope effects and rate-limiting steps involving iron and sulfur[3, 4]. Further investigation would be required to establish this.

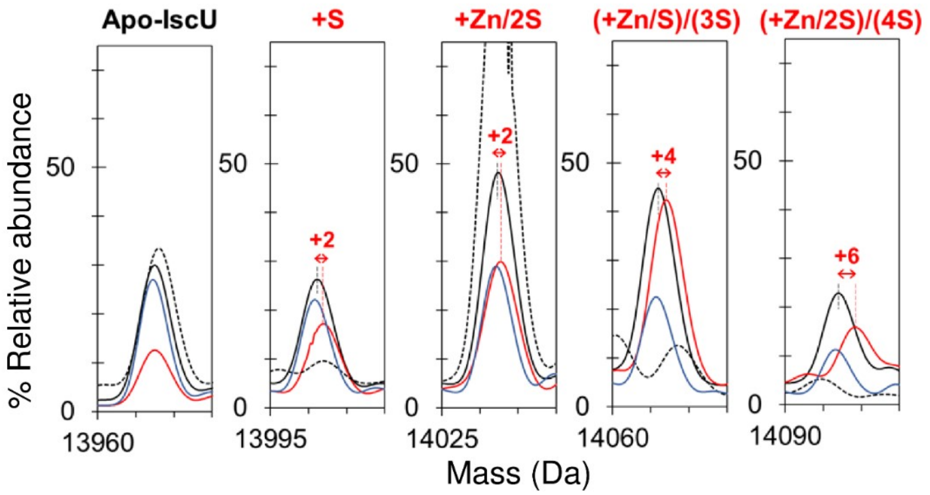

Figure S9. Detection of sulfur adducts during synthesis of [2Fe-2S] IscU monitored by discontinuous assay. Deconvoluted mass spectra of apo-IscU (10 μM containing 0.1 Zn^{2+} per protein) in 50 mM Tris, 150 mM NaCl, pH 8.0 following reconsititution and rapid exchange into 200 mM ammonium formate pH 8.0. Reconstitutions were with ^{56}Fe and ^{32}S–Cys (black line), ^{57}Fe and ^{32}S–Cys (blue line), and ^{56}Fe and ^{34}S-Cys (red line). Mass spectra of IscU prior to reaction are shown (black dotted line). Panels correspond to apo-IscU and higher mass regions where significant adducts are observed. Data from Fig. 10 of the main paper. For the three right hand panels, peaks were shifted for ^{34}S-containing samples, but the magnitude of the shift was not that expected for adducts due to bound S atoms alone. For the +96 and +128 Da peaks (natural abundance S sample), the observed shifts were +4 and +6 Da, respectively (^{34}S sample). This suggests that they are due to mixtures of Zn^{2+}/S adducts. Zn + S would give a shift of +2 Da, while 3S would give +6 Da. The observed +4 Da shift is consistent with an equal proportion of both. For the +128 Da peak, Zn + 2S would give a shift of +4 Da, while 4S would give +8 Da. Again an observed shift of +6 Da suggests an overlapping mix of the two. As described in the main paper, the stability and increased ionisation efficiency imparted by Zn^{2+}-binding accounts for the detection of these species despite their relatively low abundance compared to Zn^{2+}-free forms of IscU.

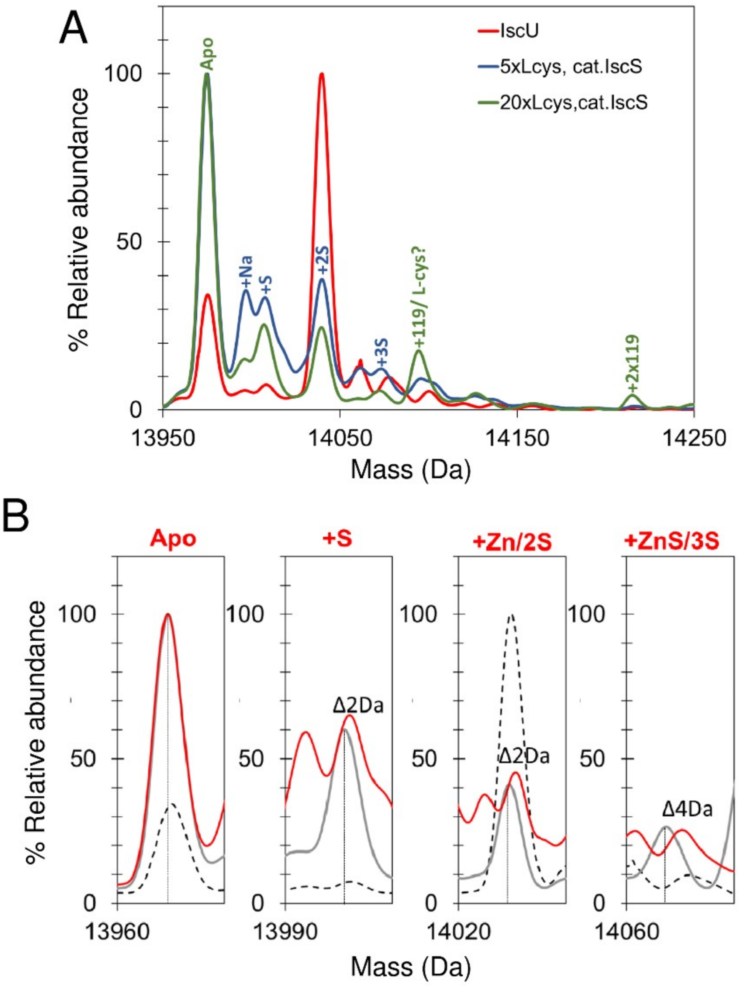

Figure S10. Detection of S adducts of IscU in absence of Fe^{2+}. (A) IscU (<0.01 Zn per protein) was incubated in ammonium formate buffer with catalytic amounts of IscS (50:1 ratio IscU:IscS) and increasing amounts of cysteine (no cysteine, red line; 5-fold excess, blue line; 20-fold excess, green line) and catalytic concentration of IscS for 30 min before infusing into the MS. (B) Incubation of IscU (black dotted line) in 200 mM ammonium formate, with IscS at a 2:1 ratio, and a 20-fold excess ^{32}S-L-cysteine (grey line) or ^{34}S-cysteine (red line).

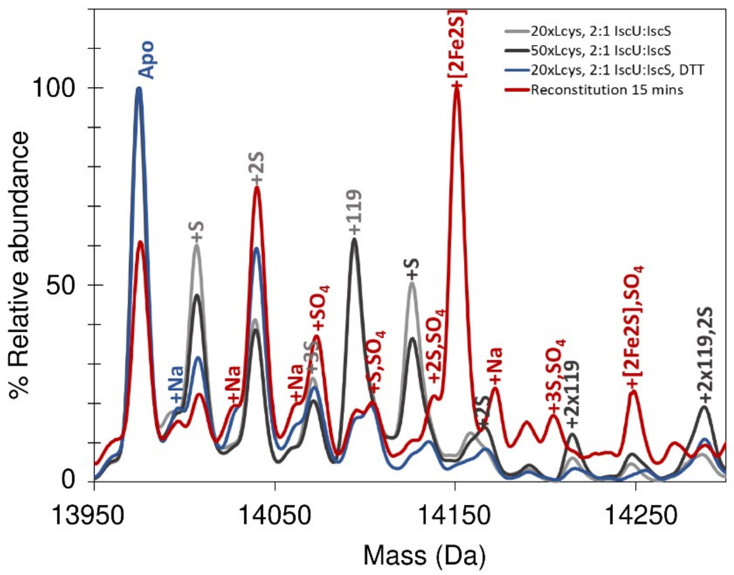

Figure S11. Reconstitution of [2Fe-2S] IscU at high concentrations of cysteine and IscS. IscU (<0.01 Zn per protein) was incubated in ammonium formate (200 mM) buffer with IscS at a 2:1 ratio, and a 20- or 50-fold excess of L-cysteine (light and dark grey lines, respectively). Addition of DTT to the 20-fold excess cysteine mixture gave the spectrum in blue, while the additional inclusion of 10-fold excess of Fe^{2+} (with all components necessary for cluster reconstitution) resulted in the spectrum in red 15 min post addition of Fe^{2+}.

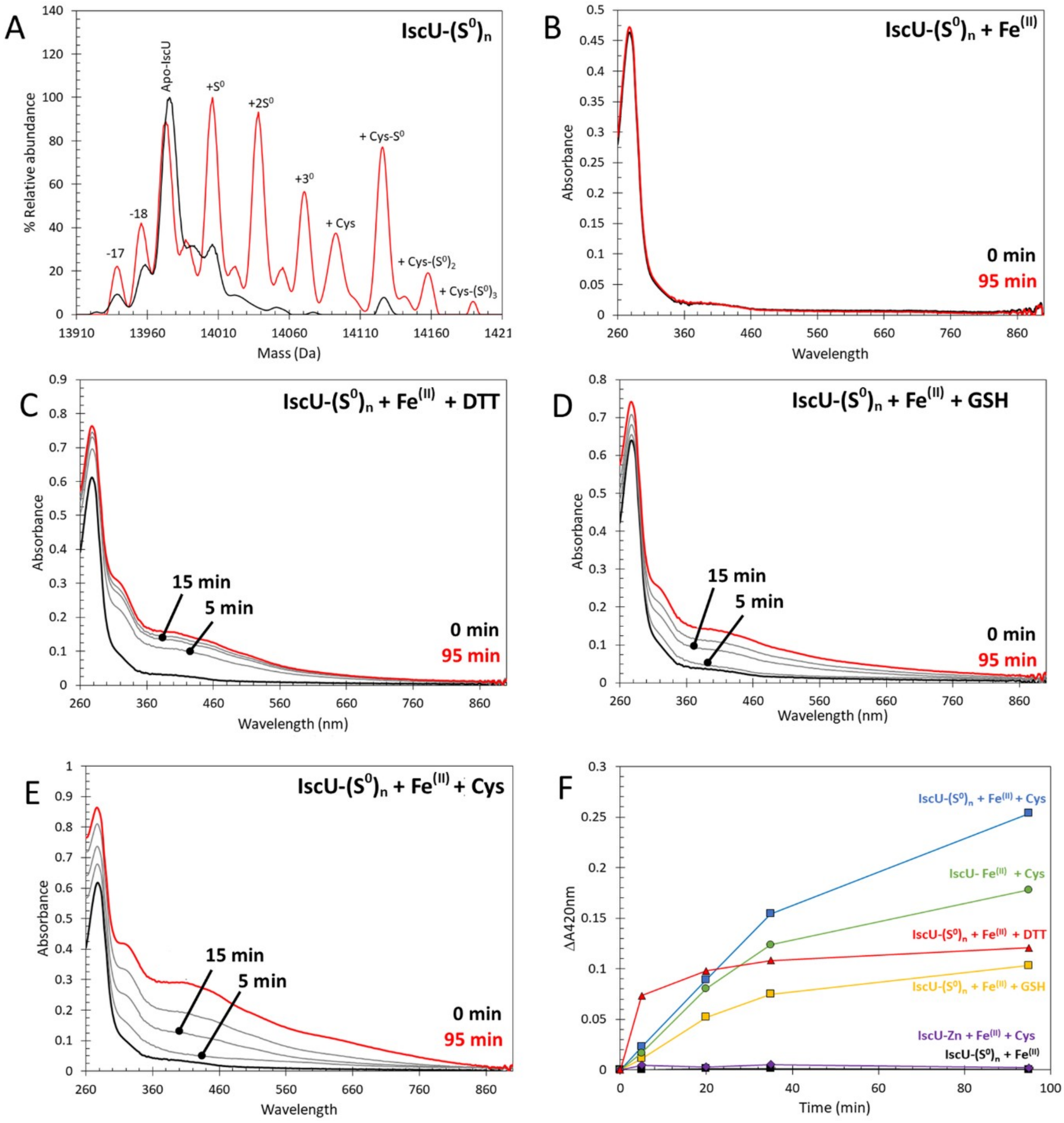

Figure S12. Assembly of a [2Fe-2S] cluster on IscU under a range of conditions. (A) LCMS of IscU before (black line) and after (red line) IscS-mediated S^0 loading. Multiple S^0 adducts were present, with ~70% IscU containing at least one S^0 per protein, in line with previous reports [1]. The ability of IscU pre-loaded with persulfide (IscU-$(S^0)_n$) to support FeS cluster assembly in the presence of (B) 0.17 mM Fe^{2+} ions, (C) Fe^{2+} ions and 2 mM DTT, (D) Fe^{2+} ions and 2 mM GSH, (E) Fe^{2+} ions and 2 mM L-cysteine. Reactions were monitored over the course of 95 min (red spectra) for [2Fe-2S] formation. (F) Change in FeS cluster absorbance ($\Delta A_{420\ nm}$) during the reactions (B – E, and Fig. 8D and E), as indicated. The persulfide form of IscU supports FeS cluster assembly in the presence of physiologically relevant low molecular weight thiols such as L-cysteine and GSH, and in the presence of DTT, a low molecular weight thioredoxin mimic.

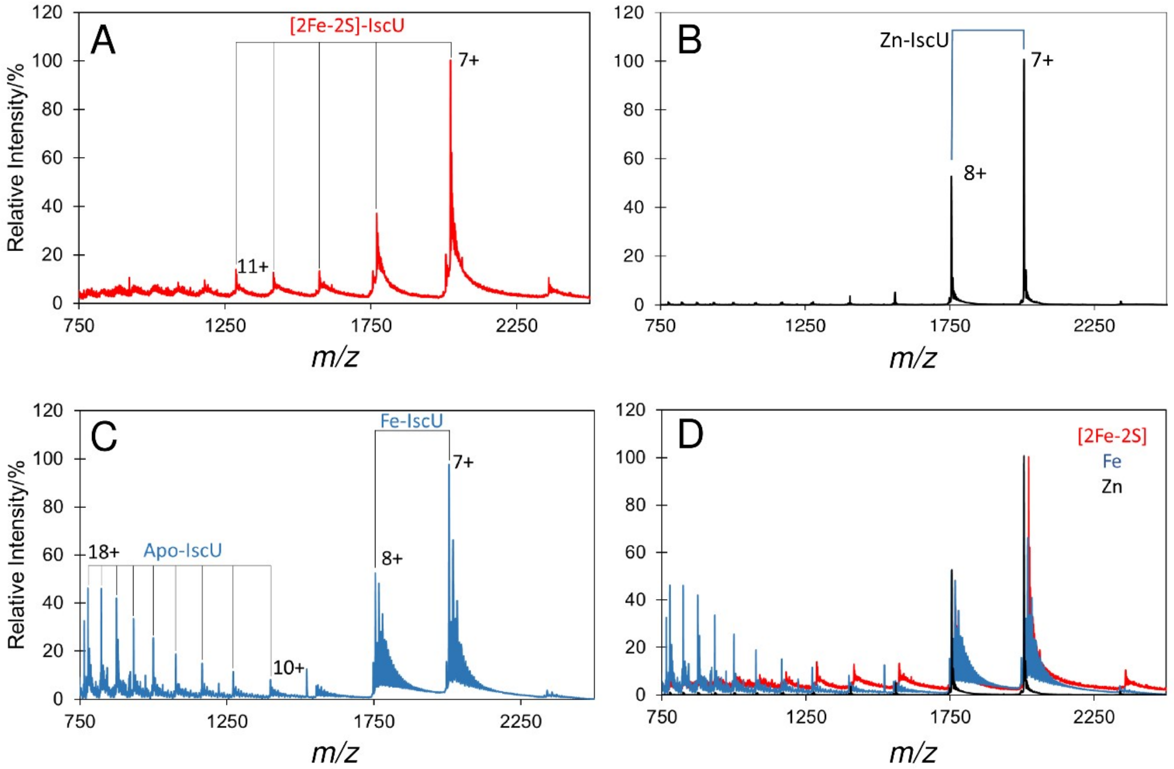

Figure S13. *m/z* spectra of IscU in [2Fe-2S], Zn^{2+} and Fe^{2+}-bound forms. (A) m/z spectrum of [2Fe-2S] IscU (repeated from Figure 4 of the main paper for comparison). (B) and (C) *m/z* spectra of Zn^{2+}- and Fe^{2+}-bound forms of IscU. (D) Overlaid plot of *m/z* spectra in (A), (B) and (C).

Supplementary References

1. M. Nuth, T. Yoon and J. A. Cowan, *J Am Chem Soc*, 2002, **124**, 8774-8775.
2. A. V. Rodrigues, A. Kandegedara, J. A. Rotondo, A. Dancis and T. L. Stemmler, *Biometals*, 2015, **28**, 567-576.
3. H. Gu and S. Zhang, *Molecules*, 2013, **18**, 9278-9292.
4. N. Nakai and M. L. Jensen, *Geochim Cosmochim Ac*, 1964, **28**, 1893-1912
